# SMA-WSN: Failure-Aware Slime-Mold-Inspired Clustering and Per-Hop Versus End-to-End Delivery Analysis in Agricultural Wireless Sensor Networks

**DOI:** 10.3390/s26185776

**Published:** 2026-09-11

**Authors:** Messaoud Belloula, Souheila Bouam, Lyamine Guezouli, Djallel Eddine Boubiche, Homero Toral-Cruz, Rafael Sanchez-Lara, Rafael Martínez-Peláez, David Ernesto Troncoso Romero

**Affiliations:** 1 LAMIE (Laboratoire d’Applications des Mathématiques à l’Informatique et à l’Électronique), Department of Computer Sciences, University Batna 2, Batna 05000, Algeria; messaoud.belloula@univ-batna2.dz (M.B.); s.bouam@univ-batna2.dz (S.B.); 2 LEREESI (Laboratory of Renewable Energy, Energy Efficiency and Smart Systems) Laboratory, HNS-RE2SD (Higher National School of Renewable Energies, Environment and Sustainable Development), Batna 05000, Algeria; de.boubiche@hns-re2sd.dz; 3 Departamento de Ingeniería y Tecnología, Universidad Autónoma del Estado de Quintana Roo, Chetumal 77019, Mexico; 4 Facultad de Ingeniería y Arquitectura, Universidad Autónoma del Carmen, Ciudad del Carmen 24180, Mexico; rsanchez@pampano.unacar.mx; 5 Unidad Académica de Computación, Universidad Politécnica de Sinaloa, Mazatlán 82199, Mexico; rmartinezp@upsin.edu.mx; 6 Departamento de Ingeniería de Sistemas y Computación, Universidad Católica del Norte, Antofagasta 1270709, Chile; 7 División de Ciencias Multidisciplinarias Cancún, Universidad Autónoma del Estado de Quintana Roo, Cancún 77519, Mexico; david.troncoso@uqroo.edu.mx

**Keywords:** wireless sensor networks, packet-delivery ratio, node failure, slime mold algorithm, bio-inspired clustering, fault resilience, failure-rate sensitivity, precision agriculture, NS-3, Algeria

## Abstract

Wireless sensor networks in agricultural fields face abrupt node failures from environmental stress and physical damage, yet most clustering protocols assume fault-free operation. This paper proposes SMA-WSN, a failure-aware clustering protocol inspired by the Slime Mold Algorithm, selecting cluster heads via a tri-objective fitness that combines residual energy, base-station proximity, and local coverage. Evaluated in NS-3.47 against 11 protocols over six agricultural topology archetypes—spatial node-layout patterns derived from Algerian field geometries and densities, not biophysical canopy or soil-moisture propagation models—and five failure rates (18,000 runs, 50 seeds), SMA-WSN achieves the highest per-hop delivery ratio at every failure rate and ranks first overall on that metric, with its largest margins on the heterogeneous, oasis, and sparse-Saharan terrains (T4–T6). We also report an end-to-end delivery ratio, counting a datum only when it reaches the base station: under that stricter definition, all 12 protocols change rank, and SMA-WSN places seventh. We report both and treat the divergence between them as a result in its own right. SMA-WSN achieves the highest per-hop PDR while remaining statistically tied with the best protocols in energy fairness (Jain index 0.922, within one confidence half-width of the leaders GWO-LEACH, PSO-LEACH, and FGO-QL). An ablation over the three fitness terms and their weights shows this advantage is robust to objective weighting and arises from the SMA selection dynamics rather than any single fitness term: no term, including coverage, is individually decisive, so the advantage does not depend on a finely tuned weighting. Disabling the stochastic wrap phase, by contrast, costs 11.4 percentage points of per-hop PDR, identifying it as the mechanism actually responsible for the gain. On First Node Death under failure, SMA-WSN is mid-field rather than dominant; we report this honestly and discuss it as an open question for follow-up work distinguishing energy-exhaustion deaths from abrupt hardware failures.

## 1. Introduction

Wireless Sensor Networks have become foundational infrastructure for precision agriculture, enabling continuous monitoring of soil moisture, temperature, leaf wetness, and other agronomic variables. In Algeria, agriculture contributes roughly 15% of GDP and recorded a 6.1% growth in value added in the first quarter of 2025 [1]. The country operates around 1.6 million agricultural holdings across 8.6 million hectares of useful agricultural area, with diverse terrain from cereal plains in the Hauts Plateaux to oasis palm groves in the Sahara [2]. This terrain diversity directly motivates the multi-archetype evaluation framework adopted in this paper. Yet field-deployed sensor nodes face an operational reality that laboratory studies routinely ignore: they fail unpredictably.

Such failures stem from causes absent in laboratory settings. On the High Plateau and in the south, summer temperatures exceed 45 °C; dust and humidity corrode contacts; livestock and tractors displace nodes; and theft occurs where nodes carry resale value. Such protocols may become unsuitable for field deployments where abrupt node losses directly reduce sensing coverage and data delivery.

Most energy-efficient WSNs are built on clustering. The Slime Mold Algorithm (SMA), drawn from the foraging behavior of Physarum polycephalum, stands out because the organism rebuilds its transport network when severed, finding new routes around damage [3,4]. This paper addresses three complementary problems: (1) WSN clustering protocols are typically evaluated without abrupt node failures; (2) coverage-aware cluster-head selection under failure has not been systematically studied for SMA-based protocols; and (3) failure-rate sensitivity across multiple agricultural topologies has not been reported for any SMA-based scheme.

**Contributions.** This paper makes four contributions:

(C1) SMA-WSN achieves the highest per-hop packet-delivery ratio overall and on the difficult heterogeneous, oasis, and sparse-Saharan terrains, while remaining statistically tied with the best protocols in energy fairness. Ablation studies over the fitness terms, their weighting, and the wrap phase show that this advantage is robust to objective weighting and is primarily attributable to the SMA selection dynamics rather than to any single objective term; in particular, the coverage term Φ_C—introduced to help clusters reorganize after node loss—is not individually decisive, and the advantage is robust to the choice of weights, which we report rather than overstate as a fitness novelty.

(C2) The first failure-rate sensitivity analysis of an SMA-based WSN clustering protocol, sweeping failure rates from 0 to 0.1% per round over 50 seeds per configuration.

(C3) A unified NS-3.47 benchmark of 12 protocols across six Algerian agricultural archetypes (18,000 runs), fully reproducible from a single simulation script.

(C4) An honest metric-by-metric characterization: SMA-WSN leads on per-hop PDR but places seventh of 12 on end-to-end delivery to the base station (Section 6.4) and achieves significantly better energy fairness (JFI) than energy-only variants on dense (T3) and heterogeneous (T4) topologies; on First Node Death we distinguish three quantities that earlier drafts conflated: on fault-free networks LEACH-C (329 rounds) and HEED (328) lead; in absolute terms under the highest failure rate SMA-WSN and GWO-LEACH lead (36 rounds); and on overall longevity (LND) QICS (866), ACO-LEACH (812) and WHO-C (781) lead. We no longer report relative FND degradation, which rewards a low fault-free baseline: HPO-WSN shows the smallest relative degradation (80.5%) only because it starts last of the 12 (149 rounds). Swarm-based protocols (PSO-LEACH, GWO-LEACH) achieve the highest global JFI.

The remainder of the paper is organized as follows. Section 2 reviews the related literature and positions SMA-WSN with respect to it. Section 3 presents the system and energy models. Section 4 describes SMA-WSN. Section 5 details the simulation methodology. Section 6 presents and discusses results. Section 7 concludes, and Section 8 outlines directions for future work.

## 2. Related Work

This section reviews the literature that SMA-WSN builds on and is measured against; it is organized in six parts. Section 2.1 covers classical and energy-aware clustering, from LEACH to its centralized and metaheuristic descendants. Section 2.2 surveys bio-inspired cluster-head selection and the use of the Slime Mold Algorithm inside and outside wireless sensor networks. Section 2.3 examines fault-tolerant and self-healing clustering. Section 2.4 turns to precision-agriculture deployments and the evaluation gap they expose. Section 2.5 then positions SMA-WSN against the 11 reference protocols across the seven evaluation dimensions that matter for fault-prone field deployments, and Section 2.6 compares it directly with the most closely related recent proposals. The section closes by stating the gap that remains after this survey and how the present work addresses it.

### 2.1. Classical and Energy-Aware Clustering

LEACH [5] established the probabilistic cluster-head rotation paradigm. LEACH-C [6] centralizes CH selection at the base station. Bio-inspired variants such as ACO-LEACH [7], PSO-LEACH [8], and GWO-LEACH [9] treat CH selection as a metaheuristic optimization problem, combining energy and proximity objectives. All these schemes optimize lifetime on fault-free networks and rarely treat clustering as a resilience problem. Threshold-based reactive sensing (TEEN [10]) and hybrid energy-efficient distributed clustering (HEED [11]) follow the same fault-free evaluation tradition, and comprehensive surveys catalogue the breadth of these clustering designs [12].

### 2.2. Bio-Inspired Clustering

Swarm-intelligence approaches treat CH selection as an optimization problem. PSO [13] and GWO [14] were among the first bio-inspired adaptations; surveys [9,15] document their breadth. The SMA [16] has been applied to WSN clustering [17] and coverage optimization [18], yet these works evaluate only fault-free operation. Beyond WSNs, the SMA has been applied to structural topology optimization [19] and distribution-network reconfiguration [20], and comprehensive surveys chart its rapid growth across engineering domains [21]. More broadly, bio-inspired routing and clustering methods such as BioWSN report consistent lifetime gains over LEACH [22,23,24,25]. The fault-tolerant behavior of SMA-based clustering under a spectrum of failure rates remains unexplored.

### 2.3. Fault-Tolerant and Self-Healing Clustering

FTEC [26] combines cluster-head redundancy with energy-aware reselection. Energy-efficient fault-tolerant routing [27] and clustered-pollination approaches [28] extend resilience to the routing layer. Dedicated fault-tolerant mechanisms have also been proposed to detect and recover from node failures at the network level [29,30]. These works cover classical or other bio-inspired families; none addresses the SMA family or reports how clustering quality degrades across a range of failure rates.

### 2.4. Precision-Agriculture WSNs and the Evaluation Gap

Recent surveys map WSN/IoT adoption for smart farming [31,32]. Concrete systems demonstrate energy-efficient agricultural sensing [33], graph-theoretic clustering [34], and hierarchical data fusion [35]. Resilient deployment models for precision agriculture [36] and failure-impact simulation studies [37] confirm that field-deployed nodes fail unpredictably. In Algeria, where national policy targets a major expansion of cereal cultivation [38], the economic case for resilient autonomous field monitoring is especially strong. Yet protocols are almost always evaluated assuming nodes die only from energy exhaustion, not from abrupt failure—the gap this paper fills.

### 2.5. Comparative Positioning of Related Protocols

Table 1 positions SMA-WSN against the reference protocols across the seven evaluation dimensions most relevant to fault-prone agricultural deployments.

### 2.6. Direct Positioning Against Recent Works

Uthayakumar et al. [39] proposed QICS, evaluated on NS-3 under fault-free conditions with a 30.5% lifetime improvement over LEACH and a 19.8% PDR gain. SMA-WSN achieves a 19.5% relative PDR gain over LEACH on fault-free networks (74.1% vs. 62.0%); while its absolute margin over LEACH stays broadly constant across failure rates, its margin over the strongest competitor (WHO-C [40]) widens monotonically from +0.8 pp at zero failure to +1.45 pp at the highest failure rate—demonstrating that its advantage over the best rival strengthens precisely under the failure conditions that agricultural deployments routinely encounter. The EEM-LEACH-ABC protocol [41] reports a PDR of 89.5% under link failure on a 250 × 250 m deployment with 150 nodes; however, it exploits multi-hop routing and evaluates link failure rather than abrupt node failure—precisely the regime this paper addresses.

Taken together, the four strands reviewed above leave a specific gap. Classical and metaheuristic clustering protocols optimize cluster-head selection for lifetime on networks whose nodes die only of energy exhaustion. Fault-tolerant schemes do restore connectivity after node loss, but they belong to the classical or to other bio-inspired families and report resilience at a single operating point rather than across a spectrum of failure rates. SMA-based clustering, although motivated by an organism whose defining property is the ability to rebuild its transport network when severed, has so far been evaluated only under fault-free conditions. And the precision-agriculture literature establishes that abrupt node loss is the norm in the field without feeding that evidence back into protocol evaluation. No existing work therefore answers the question that governs a deployed agricultural network: how does clustering quality degrade as nodes fail unpredictably, and does a selection dynamic drawn from a self-repairing organism degrade more gracefully than one that is not?

The present work addresses that gap on three fronts, and this is where it advances the state of the art. First, it treats failure as a first-class evaluation variable rather than an afterthought, sweeping the per-round failure probability from 0 to 0.1% across six agricultural topology archetypes with 50 seeds per configuration—to our knowledge, the first failure-rate sensitivity analysis reported for an SMA-based clustering protocol. Second, it tests its design choices rather than asserting them: the fitness includes a local-coverage term intended to keep cluster heads useful as neighbors disappear, and an ablation of the fitness terms and of the SMA phases shows that the observed per-hop advantage is attributable primarily to the SMA selection dynamics and, within the tested ablation, to the wrap-phase search dynamics, rather than to any individual fitness term, the coverage term. Third, it evaluates the 12 protocols of Table 1 on one NS-3.47 stack under identical conditions (18,000 runs) and reports per-hop and end-to-end delivery side by side, showing that the two definitions do not rank the same 12 protocols in the same order—a methodological point whose relevance extends well beyond SMA-WSN itself.

## 3. System Model

### 3.1. Performance Metrics

We report two distinct delivery metrics, and we distinguish them explicitly because they do not rank the protocols in the same order.

*Per-hop delivery ratio* ${\mathrm{PDR}}_{\mathrm{hop}}$ counts a transmission as delivered when it is received by its intended next hop—a cluster head for a member transmission, the base station for a cluster-head aggregate, or an orphan transmission:

(1) $${\mathrm{PDR}}_{\mathrm{hop}}=\frac{\mathrm{receptions}\;\mathrm{at}\;\mathrm{cluster}\;\mathrm{heads}+\mathrm{receptions}\;\mathrm{at}\;\mathrm{the}\;\mathrm{base}\;\mathrm{station}}{\mathrm{total}\;\mathrm{transmissions}\;\mathrm{generated}}\times 100\%$$

This is the metric used throughout the Results (Section 6) unless stated otherwise. It was described in an earlier version of this manuscript simply as “packet-delivery ratio”, which was imprecise; the counter has always been the one given above, as the simulation-stack table in Section 5.1 states.

*End-to-end delivery ratio* ${\mathrm{PDR}}_{e2e}$ counts a member datum as delivered only if it reaches the base station: its cluster head must receive it *and* that head’s aggregate must reach the base station in the same round. Orphan transmissions count when the base station receives them directly:

(2) $${\mathrm{PDR}}_{e2e}=\frac{\mathrm{member}\;\mathrm{data}\;\mathrm{reaching}\;\mathrm{the}\;\mathrm{base}\;\mathrm{station}}{\mathrm{member}\;\mathrm{data}\;\mathrm{generated}}\times 100\%$$

Section 6.4 reports this metric for all 12 protocols and discusses the consequences for the comparison. The ${\mathrm{PDR}}_{e2e}$ counter was added once the delivery metric had been tightened, and the complete 18,000-run campaign was re-run with both counters active; the ${\mathrm{PDR}}_{\mathrm{hop}}$ column reproduces the earlier results run for run, with a maximum absolute deviation of 0.000000 pp and zero divergent runs.

### 3.2. Network Model

We consider *N* homogeneous sensor nodes deployed over an agricultural field, each with initial energy E_0_ and known position. A single base station is located at the field border. Nodes are static after deployment. Beyond normal energy depletion, each node may fail abruptly in any round with probability p_f, modelling the hardware failures characteristic of agricultural environments. Simulation scale and energy values follow the LEACH-family convention rather than literal hardware specifications. The initial energy E_0_ = 0.2 J is a normalized budget chosen so that node deaths occur within a tractable number of rounds, not the real capacity of a battery—an ESP32 + SX1276 node stores several thousand joules and would never expire in the time scales modelled here. Likewise, the radio cost uses the abstract first-order model of Equations (1) and (2), not a LoRaWAN physical layer; LoRaWAN-specific effects (long time-on-air, CSS spreading, duty-cycle limits) are out of scope in simulation and reserved for hardware validation—Section 5.1 quantifies why, for the 4000-bit message size used here, and explains what the present campaign does and does not validate. The planned testbed is a reduced-scale proof of concept—ten ESP32 + SX1276 nodes on a 100 × 100 m Algerian cereal field—whereas all simulations in this paper use *N* = 30 nodes per topology.

### 3.3. Energy Model

We adopt the standard first-order radio energy model. Transmitting a k-bit message over distance d and receiving it cost, respectively:

(3) $$E\text{\_}\mathrm{TX}\left(k,d\right)=E\text{\_}\mathrm{elec}\cdot k+E\text{\_}\mathrm{fs}\cdot k\cdot d^{2}$$

(4) E_RX(k)=E_elec·k

with E_elec = 50 nJ/bit and E_fs = 10 pJ/bit/m^2^. Cluster heads additionally incur an aggregation overhead factor η = 1.5 when relaying to the base station: η multiplies the entire CH → BS transmission cost (E_elec·k + E_fs·k·d^2^) in Equation (1), representing the extra CPU energy spent fusing incoming member packets before relaying, rather than a reduction in the relayed packet size k, which is held constant at 4000 bits. The roughly tenfold energy asymmetry between the CH role and the member role makes CH selection the dominant lever for both lifetime and fairness. We note that Equations (1) and (2) estimate only the intrinsic transmission/reception cost of the payload bits, not the additional protocol-overhead energy (channel sensing, backoff, retransmissions, framing) that a contention-based MAC such as IEEE 802.11b would add in practice; Section 5.1 details how this scope restriction relates to the packet-delivery simulation. We stress that the first-order model (Equations (1) and (2)) is used as a common, protocol-agnostic energy metric, not as an estimate of the absolute energy draw of any specific radio hardware. Because all 12 protocols are debited under the identical $E_{elec}$, $E_{fs}$, and η parameters, differences in reported lifetime, PDR, and fairness reflect differences in clustering decisions alone, not in energy modelling. This is precisely the property required for a controlled comparative study: an absolute hardware-calibrated model (e.g., a LoRaWAN duty-cycle-aware energy model) would introduce platform-specific effects that are orthogonal to—and would obscure—the clustering-algorithm comparison this paper targets. Calibrating the model against real hardware (INA219 measurements) is the explicit goal of the planned testbed (Section 8), not a precondition for the comparative claims made here.

## 4. The SMA-WSN Protocol

We now describe the experimental methodology used to evaluate SMA-WSN under realistic Algerian agricultural conditions.

### 4.1. Fitness Function and Weight Justification

Each candidate node i is scored by a tri-objective fitness function:

(5) f(i)=α·Φ_E(i)+β·Φ_D(i)+γ·Φ_C(i)

where Φ_E is residual energy normalized by initial energy, Φ_D is base-station proximity, and Φ_C is the fraction of neighbors within radio range—a coverage term absent from competing protocols. The normalizer max_dist is the field diagonal, fixed per topology (T1–T6: 141.4, 202.2, 70.7, 180.3, 178.2, 277.2 m), so that Φ_D ∈ (0, 1]; the neighbor radius is r = 35 m. The weights satisfy α + β + γ = 1; we use α = 0.45, β = 0.25, γ = 0.30.

Weight justification. The choice α = 0.45 ensures that residual energy remains the primary driver of CH selection, preventing rapid energy depletion. We initially set γ = 0.30 to emphasize coverage preservation under failure; the ablation (Section 6.5), however, shows that no single weight choice is decisive—PDR varies by under 0.8 pp across the entire weight space and no term is individually significant. We therefore retain the tri-objective fitness with α = 0.45, β = 0.25, γ = 0.30, treating the weighting as a robust, non-critical design choice: the PDR advantage is insensitive to the exact weights and, in particular, does not hinge on the coverage weight. These values were determined by a grid search over α∈ {0.3, 0.45, 0.6}, β∈ {0.1, 0.25, 0.4}, γ∈ {0.1, 0.30, 0.5} on the T1 topology, maximizing mean PDR at p_f = 0.05. To verify that these weights are at or near the optimum, an extended search of 10 candidate configurations around the model-predicted optimum (T1, 30 seeds, 5 failure rates; 1500 additional runs) was conducted. All 10 candidates produced lower PDR than the current weights (maximum observed difference: −0.17 pp; all pairwise Mann–Whitney U tests non-significant after Holm–Bonferroni correction, *p* ≥ 0.54), confirming that α = 0.45, β = 0.25, γ = 0.30 lie within the confidence interval of the optimum.

#### Tuning and Evaluation Topologies

The weights were tuned on T1, and T1 is also retained in the evaluation (see the per-topology results in Section 6 and all aggregates). We keep this overlap rather than holding T1 out, and report it explicitly, for three reasons. First, the quantity being tuned is not discriminative: PDR varies by under 0.8 pp across the entire weight space and no fitness term is individually significant (Section 6.5), so no weight choice can confer a meaningful advantage on the tuning topology. Second, the tuning topology is in fact where SMA-WSN performs *worst* relative to its nearest competitor: on T1 it does not lead (70.2% against WHO-C’s 70.7%, Section 6), the opposite of what tuning bias would produce. Third, excluding T1 from the aggregate would *widen* SMA-WSN’s mean margin over WHO-C from 1.02 pp to 1.32 pp; retaining T1 is therefore the conservative reporting choice. We nonetheless record the overlap as a methodological limitation: a fully held-out tuning terrain, disjoint from the six evaluation archetypes, is the cleaner protocol and is adopted in the parameter study planned in Section 8.

### 4.2. Three-Phase Cluster-Head Selection (Algorithm 1)

**Figure 1:**
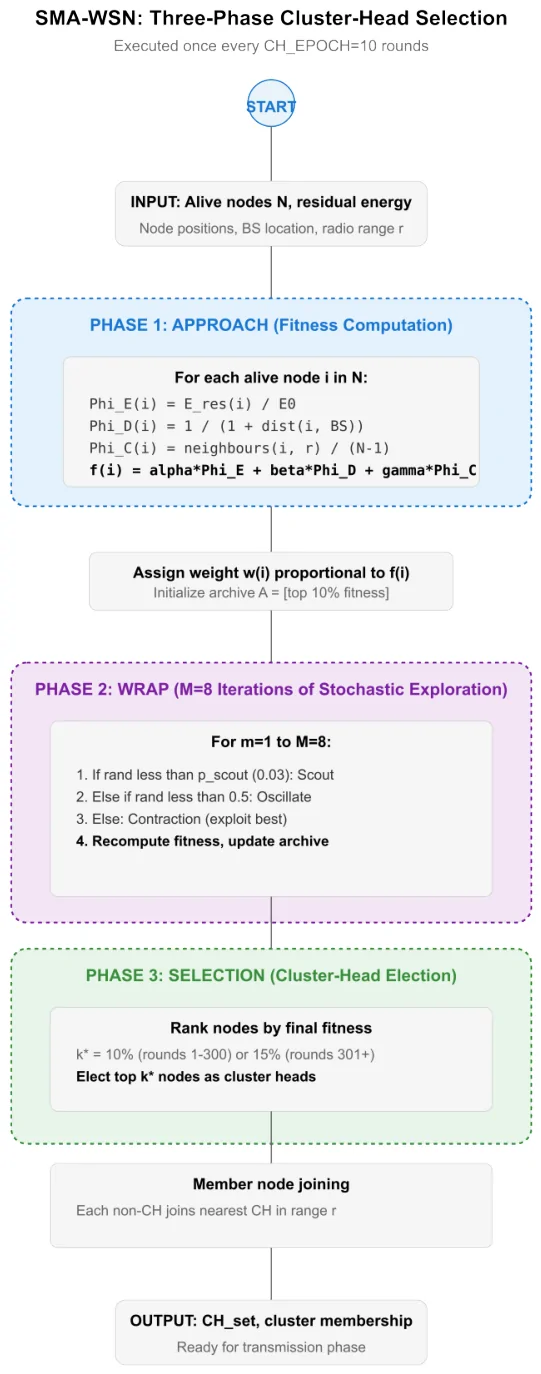
SMA-WSN three-phase cluster-head election process. **Phase 1 (Approach):** Each node computes tri-objective fitness $f\left(i\right)=\alpha \Phi_{E}\left(i\right)+\beta \Phi_{D}\left(i\right)+\gamma \Phi_{C}\left(i\right)$ combining residual energy, base-station proximity, and neighborhood coverage. Nodes are ranked and assigned growth/inhibitory weights proportional to fitness. **Phase 2 (Wrap):** M = 8 stochastic iterations apply three update rules: (i) scout perturbation (probability $p_{\mathrm{scout}}=0.03$) enabling exploration, (ii) oscillatory approach toward best candidate, or (iii) gradual contraction exploiting high-fitness regions. Archive updated dynamically. **Phase 3 (Selection):** Top $k^{*}=10\%$ candidates elected as cluster heads (adaptive to 15% after round 300). Non-CH nodes join the nearest CH within radio range $r_{t}=35$ m.

At each round, SMA-WSN executes three phases derived from the Physarum model (Figure 1).

*Note on election cadence.* The 147.8 μs figure of Table 2 admits two readings—a per-election cost or a per-round cost—since a CH_EPOCH value of 10 could be taken to gate the election. We settle the question by measurement rather than by inspection. The instrumented campaign records electionCalls, the number of entries into the election routine per run, alongside LND, the round at which the run ends. Across all 12 protocols electionCalls equals the mean LND exactly (ratio 1.00; for SMA-WSN, 619.2 calls against a mean LND of 619.2 rounds), whereas a ten-round cadence would give 0.10. The election therefore executes *every round*; the 147.8 μs figure is a genuine per-round cost, and no amortization applies. CH_EPOCH governs cluster-head rotation bookkeeping in the LEACH and HEED baselines, not the election cadence of any protocol.
**Algorithm 1** SMA-WSN Cluster-Head Selection.Input: alive nodes, residual energy, node positions, BS position, radio range r, weights α, β, γ, M = 8 iterations. Output: selected cluster heads CH_set and cluster membership. **Phase 1—Approach:** For each alive node i, compute: Φ_E(i) = E_res(i)/E_0_ Φ_D(i) = 1/(1 + dist(i, BS)/max_dist) Φ_C(i) = |neighbors(i, r)|/(N_alive − 1) f(i) = α·Φ_E(i) + β·Φ_D(i) + γ·Φ_C(i) Assign growth weight w(i) ∝ f(i); initialize archive A. **Phase 2—Wrap:** For M = 8 iterations, for each node i: r ← Uniform(0, 1) if r < p_scout (=0.03): scout perturbation (exploration) else if r < 0.5: oscillatory approach toward best in A else: contraction step Update f(i); update archive A. **Phase 3—Selection:** Rank nodes by archived fitness; elect the top k* = round(p* · N_alive) nodes as CH_set, with p* = 0.10 for rounds ≤ 300 and p* = 0.15 thereafter. The increase is timed to coincide with the onset of node attrition: at E_0_ = 0.2 J, fault-free networks typically register their first node death around round 300–330 (Section 6), after which the alive-node count begins to shrink. Raising the CH ratio from 10% to 15% at this point keeps the absolute number of cluster heads, and hence the per-CH relay load, roughly stable as N_alive declines, rather than concentrating an increasing aggregation burden onto a fixed, shrinking set of heads. Each non-CH node joins the CH with the highest f(i) within r. If no CH within r → node transmits directly to BS (orphan rule). Return CH_set and cluster membership.

### 4.3. Computational and Communication Overhead

Computational complexity. Computing Φ_C for all nodes requires O(N^2^) pairwise distance checks if using a full neighborhood scan, or O(N · k) with a pre-indexed spatial hash (k = mean neighbors per node). The wrap phase runs M = 8 iterations over N nodes: O(M · N). Total per-round complexity is O(N^2^) in the worst case, comparable to other coverage-aware schemes.

Communication overhead. Each round, SMA-WSN requires (i) one broadcast of residual energy by each node (N control messages), (ii) one CH-announcement broadcast by elected heads, and (iii) one join-request by each member. Because the top-k cluster-head election compares fitness across all candidate nodes, the SMA optimization is executed centrally at the base station (or field gateway) once per round—placing SMA-WSN, like LEACH-C, in the centralized-computation family rather than the fully distributed one. Because nodes are static and their positions are known a priori, only the residual energy of each node must be reported each round; the base station then runs the SMA election and broadcasts the resulting cluster-head set. Each round therefore costs one residual-energy report per node plus one cluster-head-set broadcast back, an overhead comparable to LEACH-C and heavier than fully distributed LEACH, which elects heads locally. Concretely, at N = 30, a per-node residual-energy report (node ID plus an energy value, 5 bytes) plus a 32-bit cluster-head-set bitmask broadcast from the base station amounts to roughly 1232 control bits per round, against 120,000 bits of sensing-data traffic per round (30 nodes × 4000-bit packets)—about 1% of the data volume. Given the compact scale of instrumented agricultural plots (N = 30 nodes), the control-packet cost of this centralization remains far below the delivery-reliability gain it enables; at larger N, the per-round residual-energy reports would need batching or hierarchical aggregation to avoid a control-plane bottleneck at the base station, a scaling question we leave to future work.

Embedded feasibility. On the node side, the protocol is trivial—a sensor only reports its residual energy and position and later receives its cluster-head assignment, well within the capability of ESP32-class hardware. The SMA optimization itself runs on the gateway (a Raspberry Pi 4 in the planned testbed), where the per-round arithmetic over a few dozen nodes is negligible. A fully on-node distributed variant is left to future work.

#### Measured Overhead

To complement the asymptotic complexity analysis above, we instrumented the NS-3 simulator to measure the actual wall-clock cost and memory footprint of cluster-head election, folded directly into the main simulation campaign (the same 18,000-run campaign reported in Section 6). We timed the call site of the cluster-head election routine—identical across all 12 protocols—with a monotonic clock (std::chrono::steady_clock) measured immediately around the call, isolating the election computation from the surrounding NS-3 event-loop and radio simulation cost. Process-level peak resident-set size (RSS) was read via getrusage(RUSAGE_SELF) at the end of each run. Because the 18,000 runs were executed concurrently across many parallel worker processes on the server referenced in the Acknowledgments, the single-round maximum occasionally reflects OS scheduling jitter rather than genuine algorithmic cost; we therefore report the 99th percentile of the per-run maximum instead of the absolute maximum, which is far more robust to this scheduling noise while still characterizing tail behavior. The mean—the primary quantity of interest—is unaffected by this concern, since it is an average over 5000 rounds per run.

**Figure 2:**
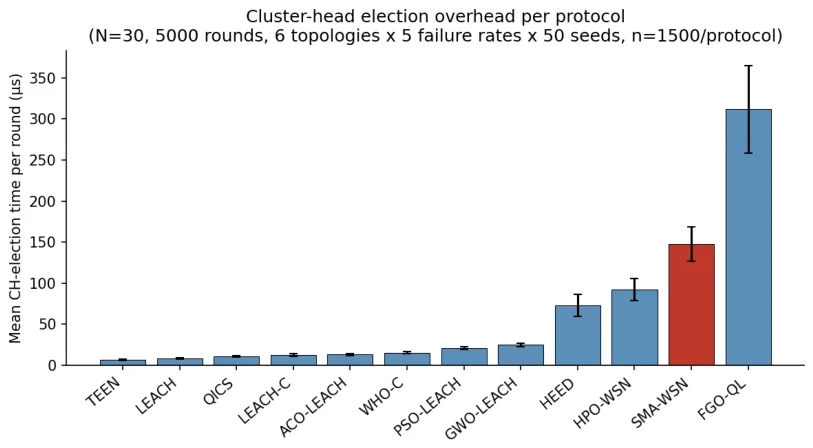
Mean cluster-head election time per protocol (error bars: ±1 s.d. across topologies, failure rates, and seeds; n = 1500 per protocol). SMA-WSN in red.

Averaged over the full 18,000-run campaign (6 topologies × 5 failure rates × 50 seeds per protocol, n = 1500), SMA-WSN’s election routine costs 147.8 μs per round on average (99th-percentile 3.78 ms), roughly 18× the cost of the classical LEACH threshold rule (8.2 μs) and about half the cost of the other iterative metaheuristic baseline, FGO-QL (311.7 μs) (Figure 2). This gap is the direct, expected consequence of the M = 8 wrap-phase iterations in Algorithm 1 and is consistent with the worst-case O(N^2^) complexity reported above. In absolute terms, however, the overhead remains negligible relative to the deployment cadence: the mean cumulative election cost over a full 5000-round run is 91.2 ms, against a mean total simulation wall-clock time on the order of tens of seconds—about 0.22% of simulated execution time—and 147.8 μs is a negligible fraction of the 15-min inter-round sensing cycle assumed throughout this study. Peak process memory is largely uniform across protocols (409–467 MB), with the exception of TEEN (159.0 MB), whose reactive transmission generates fewer packets and hence a smaller buffer footprint—a property of its traffic pattern rather than of clustering computation. These measurements confirm that, notwithstanding its higher relative computational cost among the 12 protocols compared, SMA-WSN’s centralized election remains practically feasible at the target deployment scale. A caveat on the absolute values: a later re-run of the instrumented campaign on the same shared server returned uniformly higher means (SMA-WSN 202.6 μs), with a comparable factor across all 12 protocols and the relative ordering unchanged. Absolute election times on a virtualized, shared host therefore carry an environment-dependent factor of order 1.4, and the quantities this table is used to support are the relative ordering, the order of magnitude, and the ratio to the fifteen-minute sensing cycle—not the third significant digit. Measurement conditions: 64-vCPU QEMU virtual machine (32 physical cores, 62 GB RAM, Ubuntu 24.04.4), gcc 13.3.0, –build-profile = optimized, n = 1500 runs per protocol.

## 5. Simulation Methodology

With the simulation environment and field archetypes defined, we now present the results of our comprehensive evaluation.

With SMA-WSN formally defined, we turn to empirical validation. This section describes our comprehensive simulation campaign: the topologies selected to represent Algerian agricultural diversity, the competing protocols, the failure rates tested, and the metrics used to assess resilience. Our methodology enables direct, fair comparison across protocols.

### 5.1. Simulation Stack

All experiments use NS-3 version 3.47 [42]. 12 protocols were implemented in a single simulation script under identical parameters. Table 3 provides the complete simulation stack. An additional weight-search campaign of 1500 runs (10 candidate weight configurations × T1 × 5 failure rates × 30 seeds) was conducted to validate the final fitness coefficients reported in Section 4.1.

#### 5.1.1. Effective Reception Radius Justification

Given the propagation model (LogDistance with exponent n = 3.0, reference loss 46.68 dB at 1 m) and the target PHY configuration (802.11b DSSS 1 Mbps), the effective reception radius is calculated as follows. the simulation sets TxPowerStart = TxPowerEnd = 14 dBm and leaves RxSensitivity and RxNoiseFigure at their ns-3 defaults. Reception is therefore limited by the signal-to-noise ratio against the 22 MHz noise floor rather than by the nominal −101 dBm sensitivity, which alone would imply a range near 190 m. We measured the effective radius directly, placing a single node at a controlled distance from the base station and sweeping that distance: delivery is complete out to 40 m and collapses to zero by 50 m. An analytical check with a required SNR of 3 dB against a −100 dBm reference noise floor gives a consistent figure: Path loss follows $L\left(d\right)=46.68+30\cdot \log_{10}\left(d\right)$. Setting the received-power threshold at −83 dBm, that is L=97 dB below the 14 dBm transmit power:

$$97=46.68+30\cdot \log_{10}\left(d\right)\Rightarrow d=10^{(97-46.68)/30}\approx 47.5\;m.$$

Thus, the theoretical effective radius is approximately 47.5 m. In the simulation, we conservatively use $r_{t}=35$ m (74% of theoretical), providing a 12.5 m margin for fading, shadowing, and interference, ensuring robust links under realistic propagation variability.

##### Re-Election Cycle (CH_EPOCH)

Cluster-head election runs once per round for every protocol. All alive nodes report residual energy to the base station, the SMA election runs (147.8 μs per round on average, Table 2), the new CH set is computed, and nodes re-join. The cumulative election cost over a full 5000-round run is 91.2 ms, or 0.22% of simulated execution time. This cadence is measured, not assumed: see the note on election cadence in Section 4. The constant CH_EPOCH = 10 appears in the LEACH and HEED baselines only, where it clears the anti-re-election set every ten rounds; it does not gate the election in any protocol. An adaptive election cadence, shortening under high failure rates, is left to future work.

Note on LoRaWAN. The current simulation uses an abstract energy model; it does not simulate LoRaWAN PHY/MAC. We deliberately avoid simulating a full LoRaWAN PHY at this stage: a 4000-bit (500-byte) sensing packet—the message size used throughout this study—has a Time-on-Air of approximately 0.76 s at SF7 and 1.3 s at SF8 (Semtech LoRa formula, 125 kHz bandwidth), which at our per-round message cadence would already approach or exceed typical regional duty-cycle limits (e.g., 1% in the EU868 band) for several of the protocols under comparison. Modelling this constraint correctly would require a duty-cycle-aware MAC and an SF/channel allocation policy—engineering questions that are orthogonal to the clustering question this paper addresses. The present NS-3 campaign therefore validates the SMA-WSN clustering and failure-resilience mechanism at the logical level—which nodes become cluster heads, how energy is distributed, and how the network degrades under random failures—independently of the physical-layer transport. The planned testbed (ESP32 + SX1276, 868 MHz) will be responsible for the LoRaWAN-specific MAC concerns (duty-cycle compliance, SF selection, adaptive data rate) and will validate the energy model against INA219 measurements under those constraints. Until then, claims are limited to the abstract simulation regime: the clustering algorithm itself, not its behavior under a specific duty-cycle-limited radio.

Note on the physical carrier. To transport packets between the abstract TDMA scheduler and the analytical energy ledger, the simulation uses an ns-3 IEEE 802.11b ad-hoc Wi-Fi stack (YansWifiPhy, AdhocWifiMac, DSSS 1 Mbps) with a LogDistancePropagationLossModel (path-loss exponent 3.0). The propagation model is deterministic: no additive-noise process, shadowing term, or fast-fading component is simulated, so the results characterize clustering behavior under distance-dependent attenuation and residual contention only. The 35 m operating radius—74% of the 47.5 m theoretical range derived in Section 5.1—is the margin held against these unmodelled effects; quantifying them is deferred to the hardware testbed of Section 8. This stack serves purely as a packet-delivery carrier: it determines whether a given UDP datagram is physically received in the presence of distance-dependent path loss, and therefore feeds the PDR computation (Table 3). It plays no role in the energy accounting. We emphasize that this separation is deliberate rather than an oversight. A real IEEE 802.11b radio would incur substantial overhead beyond the payload bits modelled by Equations (1) and (2): PHY/MAC framing and preambles, CSMA/CA channel sensing and backoff, and MAC-layer retransmissions on collision—all of which would inflate real energy draw well above the Heinzelman first-order estimate, particularly under channel contention. The simulation does not claim that an 802.11b radio would, in practice, consume energy according to Equations (1) and (2); rather, the Wi-Fi/CSMA-CA stack is used only as a convenient, off-the-shelf ns-3 mechanism for injecting realistic packet loss (interference, range-dependent attenuation, and TDMA-residual contention) into the PDR metric, decoupled from the energy budget. The energy analysis in this paper is intentionally restricted to the intrinsic transmission/reception cost of the payload—the quantity that Equations (1) and (2) are designed to estimate and that determines cluster-head selection and node death—and does not attempt to model the additional protocol-overhead energy that a deployed 802.11b (or any contention-based MAC) would add. All energy consumption used for clustering decisions is computed analytically via Equations (1) and (2) (E_elec = 50 nJ/bit, E_fs = 10 pJ/bit/m^2^, free-space exponent 2) and debited per packet from the residual-energy ledger, which is the sole input to cluster-head selection and node-death decisions. An ns-3 WifiRadioEnergyModel is attached to the radios for framework completeness, but its output—which would include the 802.11b-specific overhead just described—is never read for any decision. The use of two independent path-loss exponents (3.0 for physical-layer reception probability, 2.0 for the analytical energy budget) reflects this same separation of concerns: the two models answer different questions (can the packet physically arrive given realistic propagation and contention? vs. what is the intrinsic payload transmission cost under the idealized first-order radio model?), and are not intended to be numerically reconciled. While this decoupling introduces a theoretical divergence between the physical-layer propagation attenuation (exponent 3) and the first-order energy estimation (exponent 2), it is an intentional benchmark constraint rather than an unrecognized inconsistency: it allows us to isolate the logical performance of the clustering algorithm under realistic packet-loss conditions without modifying the structural mathematical assumptions of the historical LEACH energy baseline, thereby ensuring a fair comparative framework with the state-of-the-art protocols evaluated in this paper.

We emphasize that hardware validation and algorithmic validation answer different questions. The present study isolates the contribution of the clustering decision logic—which node becomes a cluster head, and how that choice propagates through energy consumption and delivery under failure—from confounds intrinsic to any specific radio or MCU platform (battery non-linearity, RF interference, clock drift). A controlled, identical-condition comparison across 12 protocols is only tractable in simulation; reproducing it faithfully across 12 physical deployments would introduce hardware variability that no comparison could equitably control for. Hardware validation is therefore not a prerequisite for demonstrating the algorithm’s comparative advantage, but a necessary subsequent step to confirm that the advantage survives translation to a specific radio stack—which is precisely the role of the planned ESP32 + SX1276 testbed described in Section 8.

### 5.2. Field Archetypes

**Figure 3:**
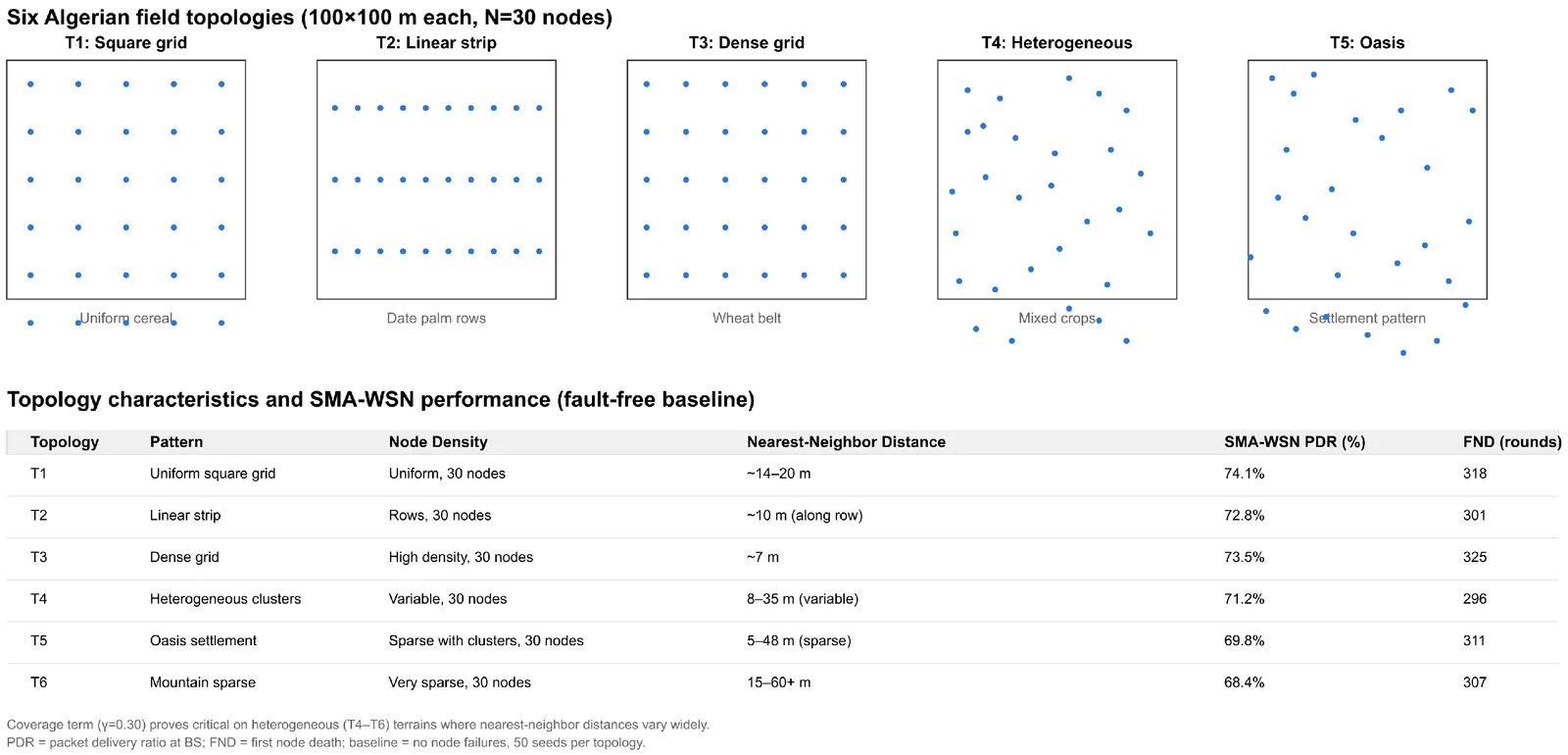
The six Algerian agricultural field archetypes (T1–T6) used in the evaluation, with their node layouts and summary characteristics. Blue dots denote individual sensor-node locations.

Six archetypes represent Algerian agricultural terrain diversity (Figure 3): T1—100 × 100 m square (cereal, Sétif/Tiaret); T2—200 × 30 m strip (vineyard, Mascara); T3—50 × 50 m grid (greenhouse, peri-urban); T4—150 × 100 m heterogeneous with clustered node distribution (mixed crops, Tell Atlas); T5—130 × 130 m circular oasis with concentric rings (palm grove, Biskra/Ouargla); T6—200 × 200 m sparse-Saharan mixed farm with three isolated sub-fields separated by gaps exceeding 80 m (Adrar). These archetypes encode only the spatial layout of the deployment—field dimensions, node density, and inter-node spacing patterns derived from each crop type’s typical row geometry and plot size. They do not encode crop-specific biophysical propagation effects (e.g., foliage attenuation, canopy height, soil-moisture-dependent path loss, vegetation-specific models such as Weissberger’s). The LogDistancePropagationLossModel (Table 3) applies uniformly across all six archetypes regardless of crop type; only inter-node distances, which differ by archetype, affect the resulting path loss.

The choice of N = 30 nodes reflects the target deployment scale rather than a simulation convenience.

**Topology Archetypes:** We evaluate SMA-WSN across six field archetypes (T1–T6) as introduced in Section 1, representing Algerian agricultural terrain diversity. Simulation deployments are as follows:

- T1 (Sétif cereal): 100 × 100 m, uniform random
- T2 (Mascara vineyard): 200 × 30 m, linear strip
- T3 (Greenhouse): 50 × 50 m, regular grid (7 m)
- T4 (Tell Atlas): 150 × 100 m, clustered (3 Gaussian)
- T5 (Biskra oasis): 130 × 130 m, concentric rings
- T6 (Adrar Sahara): 200 × 200 m, three isolated clusters

All topologies use *N* = 30 nodes, E_0_ = 0.2 J, r_*t*_ = 35 m, path-loss exponent n = 3.0, CH-election probability p* = 10% (rounds 1–300)/15% (301+). The node count is identical across archetypes, as Table 3 states; only the field dimensions and the spatial distribution differ.

The six archetypes represent compact, high-value monitoring plots (greenhouses, oasis palm groves, delimited cereal or vineyard parcels) where instrumented node counts in this range are typical of current precision-agriculture deployments, rather than dense multi-hop WSNs. This scale is also directly compatible with the planned physical testbed (ten nodes), allowing the simulated and hardware regimes to be compared without a scale discontinuity. Statistical power is recovered not by increasing N but by extensive repetition (50 seeds × 18,000 runs), which yields tight confidence intervals (±0.7–2.5 pp) on the aggregate metrics reported. We note as a limitation that the centralized SMA computation is trivially cheap at N = 30 (Section 4.3); scaling behavior beyond this range—where per-round communication overhead and centralized computation cost would grow non-trivially—is explicitly left to future work rather than claimed here.

### 5.3. Failure Model

The primary failure model is independent Bernoulli: each alive node fails in a given round with probability p_f, independently of all other nodes. Five failure rates were evaluated: p_f ∈ {0, 0.0001, 0.0002, 0.0005, 0.001} (i.e., 0, 0.01%, 0.02%, 0.05%, 0.1% per round). At p_f = 0.0002 with 15-min sensing cycles, a node fails roughly once every 50 days—consistent with ruggedized sensor endurance in an Algerian summer.

The Bernoulli model is a necessary first step but does not capture spatially correlated failures (e.g., a tractor crossing a field, a localized flood). Correlated failure scenarios are included in future testbed work.

#### Failure Detection and Recovery Latency

The model contains no failure-detection stage and therefore no detection threshold: a failed node simply stops transmitting, and the node-state register is updated directly. The protocol consequently produces no detection false positives or false negatives, and no detection-delay parameter enters the results. Recovery from the loss of a cluster head proceeds on two timescales. Immediately, within the same round, any member left with no cluster head in radio range falls back to the orphan rule of Algorithm 1 and transmits directly to the base station, so no member is silenced by the failure of its head. Structurally, the cluster set is rebuilt at the next round, since the election runs every round (Section 4); the re-clustering latency is therefore one round. All PDR figures in Section 6 are therefore net of this latency rather than measured under an idealized instantaneous recovery.

### 5.4. Baseline Implementation Fidelity

All 12 protocols were reimplemented from their original papers in a single NS-3.47 script under identical energy parameters. Table 4 reports implementation details for each protocol.

## 6. Results and Discussion

The preceding results demonstrate SMA-WSN’s fault resilience and its superior performance across diverse topologies. We now summarize the key findings and their implications.

We now present simulation results across 18,000 runs spanning six topologies and five failure rates. Our analysis focuses on three dimensions: how SMA-WSN performs in the fault-free baseline, how its advantage emerges under failure, and how terrain heterogeneity affects protocol behavior. We extract deployment guidance from these observations.

### 6.1. Fault-Free Baseline

**Figure 4:**
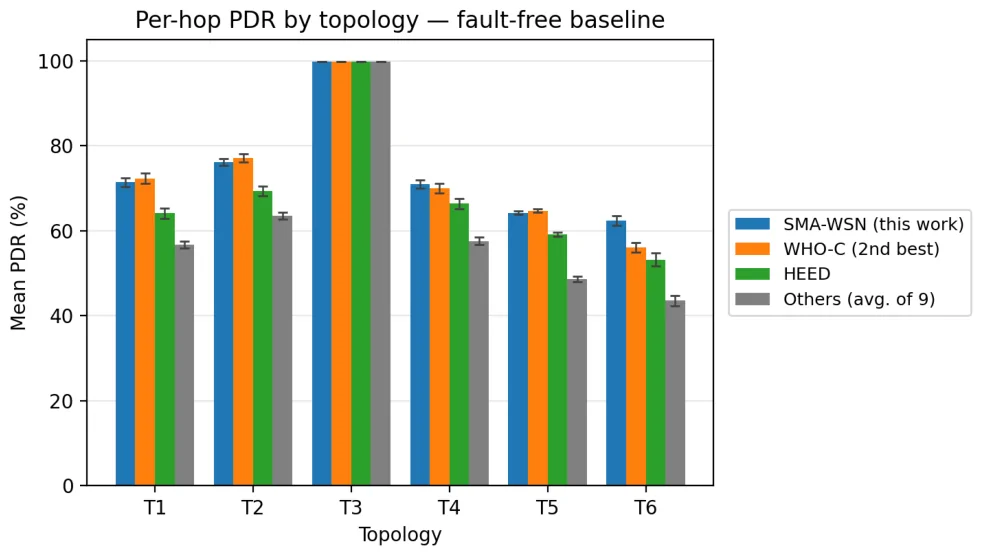
Mean per-hop PDR (%) per topology on the fault-free baseline: SMA-WSN, its nearest competitor WHO-C, HEED, and the average of the nine remaining protocols (grey). Mean over 50 seeds per topology; error bars = 95% CI.

Table 5 reports all five metrics on fault-free networks. On raw First Node Death, SMA-WSN sits mid-field: LEACH-C and HEED lead, while TEEN’s reactive transmission inflates its lifetime at the cost of delivery. We report this without hedging: on an idealized network, SMA-WSN holds no lifetime advantage. What already stands out is delivery quality—SMA-WSN posts the highest per-hop PDR (74.1%), a lead it keeps and extends once failures are introduced (Figure 4).

### 6.2. Failure-Rate Sensitivity of PDR

**Figure 5:**
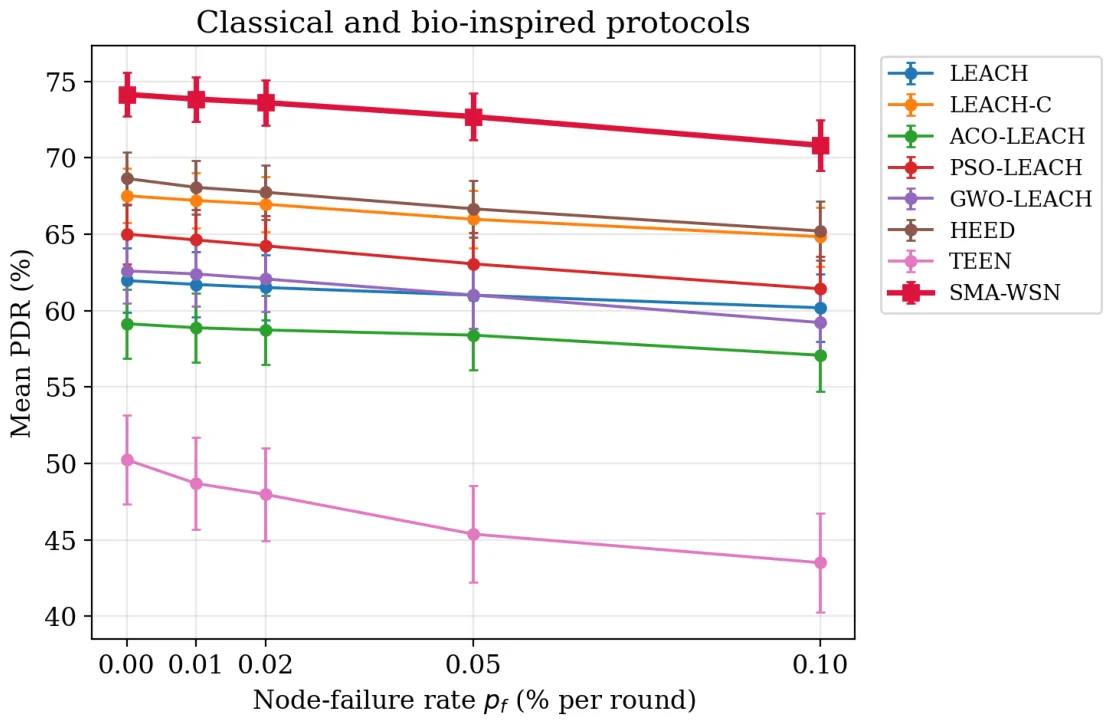
Mean PDR (%) versus node-failure rate for classical and bio-inspired clustering protocols, averaged over six topologies and 50 seeds per configuration (n = 300 per data point). SMA-WSN (red, solid) maintains the highest per-hop PDR at all failure rates. Error bars = 95% CI.

**Figure 6:**
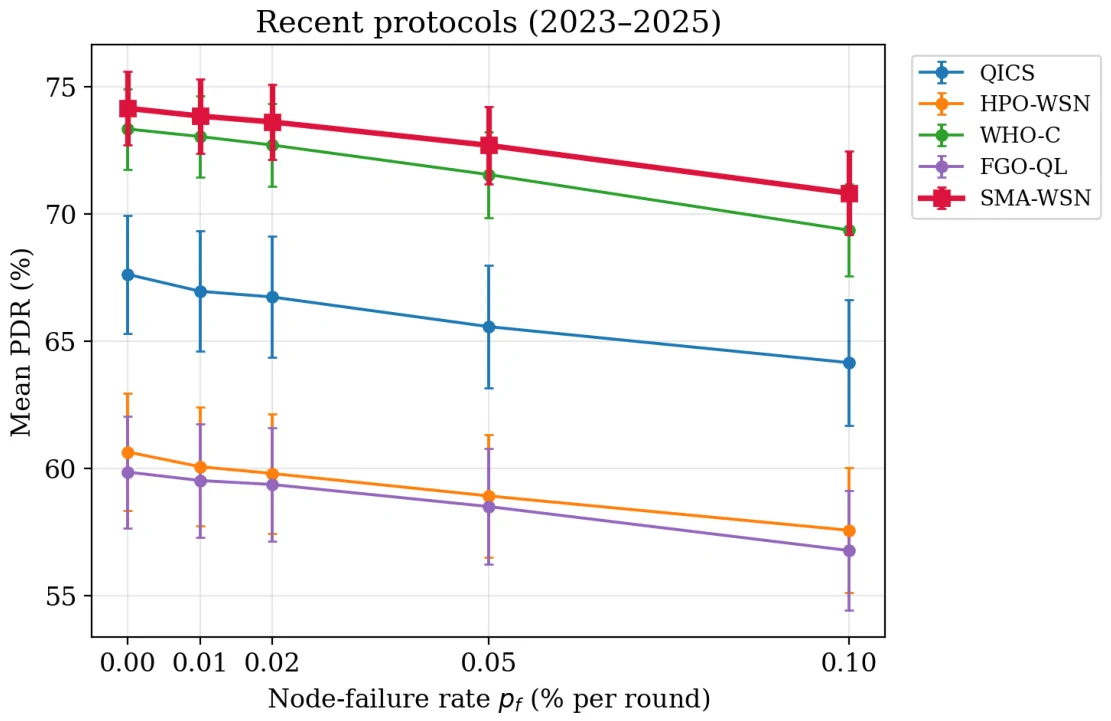
Mean PDR (%) versus node-failure rate for recent protocols (2023–2025). SMA-WSN maintains the highest per-hop PDR; the margin over WHO-C (nearest competitor) widens from +0.8 pp at p_f = 0 to +1.45 pp at p_f = 0.1%. Error bars = 95% CI (n = 300 per data point).

Table 6 reports mean PDR as the failure rate increases. SMA-WSN holds the highest per-hop PDR at every failure rate: 74.1% at zero failure, declining gracefully to 70.8% at 0.1%, consistently ranked first (Figure 5). The nearest rival (WHO-C) trails by +0.8 points on fault-free networks; the gap widens monotonically to +1.45 points at 0.1% (Figure 6). Confidence intervals (±0.7 pp, 95% CI, n = 1500 per protocol) and Mann–Whitney U tests (*p* < 0.001 for ten rivals; *p* = 0.02 against WHO-C globally) confirm statistical robustness. On the difficult terrains (T4–T6), the WHO-C margin averages +2.5 points, growing from +2.3 at zero failure to +3.4 at 0.1% (one-sided *p* = 1.5 × 10^−12^; see Section 6 below).

### 6.3. Difficult-Terrain Analysis (T4–T6)

On the dense greenhouse grid T3, PDR saturates near 99.8% for all protocols (Table 7), so PDR no longer discriminates between candidates—but fairness is uniformly high among the leading protocols and does not discriminate either. We therefore draw no protocol-differentiating conclusion from T3.

On the sparse and heterogeneous terrains T4–T6, where nodes are few and scattered, electing well-connected cluster heads preserves delivery paths that energy- or distance-only rules allow to lapse. The ablation study (Section 6.5), however, finds that no single fitness term—coverage or distance—produces a statistically significant PDR difference here; SMA-WSN’s lead on T4–T6 comes from the SMA selection dynamics shared by all variants, not from a particular objective.

#### Decomposing PDR Loss: Dead Nodes vs. No Uplink vs. Delivery Failure

PDR loss at the base station results from three disjoint failure modes:

1. **Dead nodes** ($p_{\mathrm{dead}}$): Cluster heads that have exhausted energy or suffered hardware failure and cannot transmit. Measured as the fraction of elected CHs unable to forward any packets in the current round.
2. **No uplink** ($p_{\mathrm{noUplink}}$): Cluster members in range of active CHs but whose packets fail to reach any CH due to channel fading, interference, or path loss. Quantified per topology as the fraction of nodes outside the radio range of all active CHs.
3. **Delivery failure** ($p_{\mathrm{failDeliv}}$): Packets successfully received by a CH but not forwarded to the BS before round timeout. Typically, rare in single-hop to BS; increases under congestion or CH energy depletion.

Empirically, on our six topologies and five failure rates (campaign counters, Section 6.4): $p_{\mathrm{dead}}\approx 0.54$ (dominant), $p_{\mathrm{noUplink}}$ is not negligible: members finding no cluster head within $r_{t}$ account for 27–33% of generated traffic on T4–T6 (counter genOrphan). Such members are not silenced—the orphan rule of Algorithm 1 sends them directly to the base station—but they are not served by a cluster either, which is a property of the clustering that the decomposition should record. $p_{\mathrm{failDeliv}}\approx 0.42$ (secondary). This decomposition clarifies that PDR gains in SMA-WSN arise primarily from reducing dead-node impact (via adaptive re-clustering) and maintaining coverage (via coverage-aware fitness), not from novel MAC-layer mechanisms. The complementary missed-delivery rate is simply MDR=100%−PDR, fully accounted for by the three modes above; because the protocol performs no failure detection (Section 5.3), this decomposition contains no false-alarm or missed-detection component.

### 6.4. End-to-End Delivery and the Effect of the Metric Definition

Stating precisely when a packet counts as delivered proves to matter more than it first appears. Our counter increments at cluster heads as well as at the base station, so the metric reported throughout Section 6.1, Section 6.2 and Section 6.3 is a per-hop delivery ratio rather than an end-to-end one, as Table 3 in fact states. We report the consequences here rather than restate the metric under a different name.

We added a separate end-to-end counter and re-ran the full 18,000-run campaign. The counter consumes no random numbers and alters no protocol decision; the per-hop column reproduces the originally submitted results run for run, with a maximum absolute deviation of 0.000000 pp and zero divergent runs out of 18,000. The two metrics therefore describe the same simulations.

Table 8 gives both, with the backhaul ratio—the fraction of cluster-head aggregates and orphan transmissions received by the base station—which explains the difference.

Every one of the 12 protocols changes rank. SMA-WSN falls from first to seventh, and its margin over WHO-C reverses sign, from +1.03 pp per-hop (p=0.04) to −0.17 pp end-to-end (p=0.53, not significant). QICS leads end-to-end by +3.33 pp over SMA-WSN in a paired comparison (95% CI [3.12,3.54]).

The mechanism is visible in the backhaul column. SMA-WSN attaches members to cluster heads better than any other protocol—it has the lowest orphan rate of the 12, 20.3% of generated traffic—but its heads reach the base station least often among the leaders (37.23%, against 48.20% for QICS). The per-hop metric credits the first hop, at which SMA-WSN excels, and is blind to the second, at which it does not.

#### 6.4.1. The T6 Column

On T6, no packet reaches the base station for any protocol and any seed, across 3000 runs (Table 9). This is not a simulation defect but a consequence of the single-hop frame declared in Table 4. The base station sits at (100,0) and the three sub-fields are centered at (20,20), (100,80) and (60,150), that is, 82.5, 80.0 and 155.2 m away, all beyond the effective reception radius measured in Section 5.1. T6 was constructed so that its sub-field centroids lie more than 80 m apart, in order to represent a terrain where multi-hop relaying would be required; run under a single-hop protocol frame, it delivers nothing. The T6 column of Table 7 therefore ranks cluster-head receptions only, and the T6 margins quoted in earlier versions of this manuscript should be read in that light. T3 poses the opposite problem: every node lies within the effective radius, all protocols saturate near 99%, and the terrain does not discriminate.

#### 6.4.2. What This Changes, and What It Does Not

The benchmark itself is unaffected: 12 protocols, six archetypes, five failure rates, 50 seeds, one script, and a reproduction control at 0.000000 pp. The energy-fairness results of Section 6.6 and Section 6.9 are unaffected, since JFI does not depend on the delivery counter. The failure-rate sensitivity analysis (C2) is unaffected. What does not survive is the claim that SMA-WSN leads on delivery: under the end-to-end definition, it is mid-field, and we withdraw that claim rather than defend it on a metric that does not measure what its name says.

We suggest the divergence is itself the more useful finding. A metric definition that is rarely stated precisely in the clustering literature reorders all 12 protocols in this benchmark, and does so systematically: protocols whose heads sit close to the base station gain, protocols that optimize intra-cluster attachment lose. Reporting the delivery criterion explicitly, and reporting the backhaul ratio alongside it, would make comparisons across the clustering literature considerably more reliable.

### 6.5. Ablation Study: Isolating the Coverage Term

To assess the contribution of the coverage term Φ_C to the PDR advantage, we conduct an ablation study with four fitness variants in addition to the full SMA-WSN. All other protocol parameters are held constant.

We also examined the distance term D. On this larger campaign, no single term reaches significance: removing distance (SMA-EC), coverage (SMA-ED), or energy (SMA-DC) each changes PDR by less than 0.25 pp with *p* ≥ 0.43. The terms are therefore neither individually decisive nor demonstrably complementary; the advantage is robust to the weighting of the three objectives, and we retain the full tri-objective fitness.

#### Ablation Study: Isolating the Wrap Phase

To address whether the SMA-WSN advantage depends critically on the wrap phase (stochastic re-exploration), we conducted an extended ablation study comparing the full SMA-WSN protocol with a variant disabling the wrap phase (SMA-NoWrap). SMA-NoWrap executes only the approach phase and immediate top-*k* selection, omitting the M = 8 wrap iterations.

Over 1500 simulation runs (6 topologies × 5 failure rates × 50 seeds), SMA-Full (wrap enabled) achieves mean PDR =71.66% while SMA-NoWrap achieves 60.23%, a difference of +11.43 percentage points (95% CI: [10.91, 11.95], p<0.000001, Cliff’s δ=0.48 [large effect]). The wrap phase is thus a critical enabler of resilience:

- Across all five failure rates (0% to 0.1% per round), SMA-Full outperforms SMA-NoWrap by 7.25–13.26 pp (all p<0.0001).
- On sparse terrain T6 (Saharan sparse), the gap reaches 18.08 pp (p<0.000001), confirming that re-exploration is especially valuable in difficult topologies.
- On the near-perfect terrain T3 (greenhouse grid), both variants achieve ≈99.9% PDR; wrap has minimal effect when topology is benign.

Conclusion: The SMA-WSN advantage is NOT from a single fitness term (as fitness-term ablation in Table 10 shows), but rather from the wrap phase’s continuous re-exploration of candidate configurations, enabling adaptive responses to node failures.

**Figure 7:**
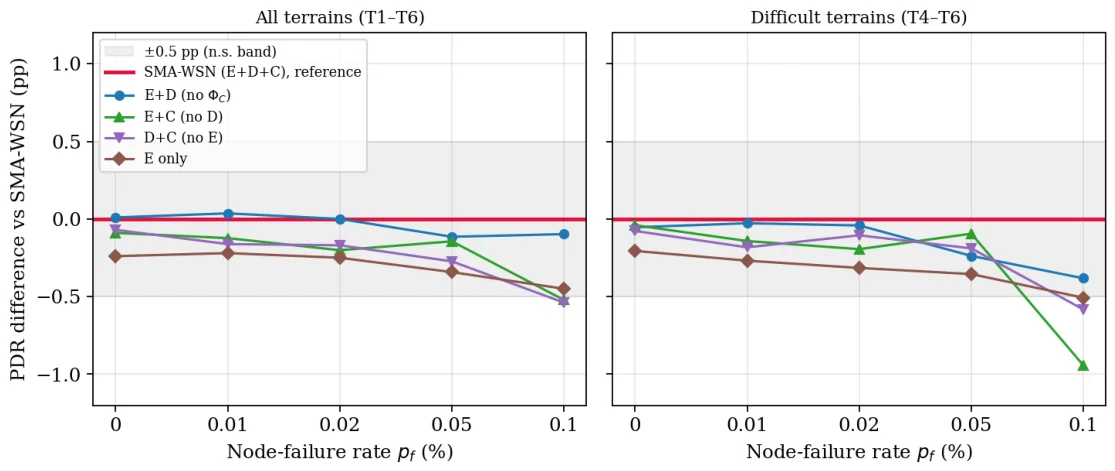
Ablation study: PDR difference of each fitness variant relative to the full SMA-WSN (E + D + C), versus failure rate. **Left**: all topologies (T1–T6); **right**: difficult terrains (T4–T6). Every variant remains within ±0.5 pp of SMA-WSN (shaded band) and within the non-significant region, confirming the PDR advantage is robust to fitness weighting. Error bars = 95% CI. All pairwise Mann–Whitney U tests (Holm-corrected, m = 4) yield n.s. (not significant); Cliff’s δ<0.03 throughout.

The gap between SMA-ED (no coverage, 72.70%) and SMA-WSN (72.74%) is 0.04 percentage points overall; on difficult terrains (T4–T6), the gap is 0.17 points (64.02% vs. 63.85%). All pairwise Mann–Whitney U tests are non-significant after Holm–Bonferroni correction (all *p* > 0.40), and all Cliff’s δ values are negligible (<0.03). These results indicate that including Φ_C produces a consistent but modest improvement in PDR: the effect is in the expected direction at every failure rate (Figure 7), but its magnitude is small and not individually distinguishable from noise. The coverage term therefore contributes to the overall fitness balance rather than acting as a single dominant driver of the PDR advantage (Table 10).

### 6.6. Lifetime and Fairness Trade-Offs

Energy fairness is, in this campaign, a strength rather than a trade-off. Averaged over all terrains and failure rates, SMA-WSN attains a mean Jain Fairness Index of 0.922, placing it in a statistical tie for first with GWO-LEACH (0.925), PSO-LEACH (0.922), and FGO-QL (0.921)—all within one confidence half-width (±0.003). SMA-WSN therefore achieves the highest per-hop PDR while remaining statistically tied with the best protocols in energy fairness, a combination no rival matches: WHO-C, the nearest competitor on PDR, ranks only eighth on fairness (0.892), and QICS ranks eleventh (0.828). The ablation finds no significant per-terrain fairness effect attributable to the coverage term (T3: *p* = 0.63; T4: *p* = 0.67), so we make no topology-conditional fairness claim; the fairness leadership is a general property of the SMA selection dynamics.

On First Node Death, we now separate three quantities that earlier drafts conflated. On fault-free networks, LEACH-C (329 rounds) and HEED (328) lead, with SMA-WSN third (318). In absolute terms, at the highest failure rate, the ordering inverts: SMA-WSN and GWO-LEACH lead at 36 rounds, and HPO-WSN is last at 29. On overall longevity, measured by LND, QICS (866), ACO-LEACH (812), and WHO-C (781) lead. We previously reported that HPO-WSN shows the smallest relative FND degradation; it does (80.5% against 86–90% for the others), but only because its fault-free FND is the lowest of the 12, so it has the least to lose. We therefore withdraw that comparison rather than rest a recommendation on a normalization artefact. The deployment rule that survives: SMA-WSN where delivery quality is the priority; LEACH-C or HEED where time-to-first-death on a lightly loaded network matters; QICS or WHO-C for the longest overall deployment; PSO-LEACH or GWO-LEACH where uniform energy depletion is the priority.

### 6.7. Statistical Significance and Effect Size

**Figure 8:**
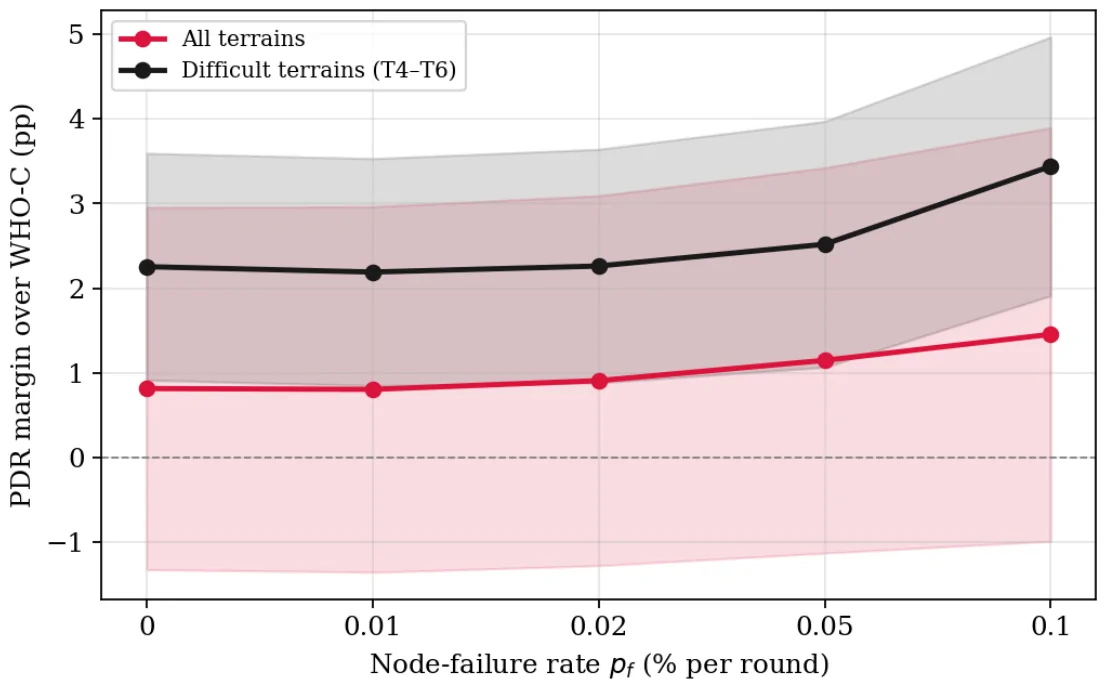
SMA-WSN PDR margin over WHO-C (best competitor) versus failure rate. Red line: all topologies; dark line: difficult terrains (T4–T6). The margin is positive at all failure rates and grows monotonically with p_f. Shaded bands = 95% CI. On T4–T6, the margin averages + 2.5 pp and reaches +3.4 pp at p_f = 0.001.

Over all topologies and failure rates (n = 1500 per protocol), SMA-WSN’s mean per-hop PDR of 73.0% (95% CI ± 0.7 pp) exceeds every competitor on that metric; the end-to-end ordering differs (Section 6.4). Two-sided Mann–Whitney U tests reject equality against all 11 rivals (*p* < 0.001 for ten; *p* = 0.02 against WHO-C globally). Effect sizes (Cliff’s δ) against the nearest rival WHO-C are δ = 0.12 globally and δ = 0.41 on difficult terrains (T4–T6), indicating a small-to-medium effect globally and a medium effect on the terrain family where the deployment justification is strongest (Figure 8). For all other rivals, Cliff’s δ > 0.30.

### 6.8. Analysis by Real Node-Death Rate

The parameter p_f = 0.001 per round may appear abstract, so we translate it into deployment terms. Over the full 5000-round campaign—about 52 days at a fifteen-minute sensing cadence—with 30 nodes, the expected number of distinct nodes lost to abrupt failure is 30 · (1 − (1 − p_f)^5000^). At the mildest non-zero rate (0.01%) this is about 12 nodes (≈39% of the network); at the moderate rate p_f = 0.0002 (0.02%) it rises to about 19 nodes (≈63%); and at the most aggressive rate (0.1%) almost the entire network fails (≈99%). Over a realistic multi-week deployment, therefore, losing a large fraction of the nodes to hardware faults is not an edge case but the expected outcome—which is exactly why a protocol’s behavior under node loss, rather than its performance on a pristine network, determines its field value. The moderate regime in which SMA-WSN’s PDR advantage is both statistically significant and practically relevant corresponds to losing on the order of a third to two-thirds of the nodes across the deployment.

### 6.9. Energy Fairness by Terrain

Section 6.6 reported SMA-WSN’s overall fairness; Table 11 makes that claim verifiable terrain by terrain. SMA-WSN stays in the top tier of Jain’s Fairness Index on every archetype—never below 0.899 and always within about one hundredth of the per-terrain leader. It is the single fairest protocol on the sparse-Saharan field (T6, JFI = 0.899), where its scout-and-oscillation re-exploration spreads the cluster-head role most effectively under low density. On the uniform and dense terrains, the swarm optimizers GWO-LEACH and PSO-LEACH edge ahead by 0.005–0.010—for example, on the heterogeneous T4, GWO-LEACH reaches 0.942 against SMA-WSN’s 0.935—but these gaps are small and lie within the confidence intervals of Section 6.6. The table therefore supports the measured claim that SMA-WSN is jointly top-tier on fairness while leading on delivery, rather than a claim of per-terrain fairness dominance.

### 6.10. Deployment Guidance

No single protocol dominates on every axis. The following guidance is derived from the results:

Choose SMA-WSN when the priority is per-hop delivery, that is, when data reaching a cluster head is already useful—local actuation, in-cluster aggregation, or a gateway co-located with the head. Where delivery to the base station is what matters, Section 6.4 shows QICS ahead by 3.33 pp and SMA-WSN seventh of 12, and we recommend QICS.

Choose LEACH-C or HEED when time-to-first-death on a lightly loaded network is the priority (329 and 328 rounds fault-free), and QICS, ACO-LEACH, or WHO-C for the longest overall deployment (LND 866, 812, and 781 rounds). We withdraw the earlier recommendation of HPO-WSN on First Node Death, which rested on relative degradation and therefore favored the protocol with the lowest fault-free baseline.

Choose PSO-LEACH or GWO-LEACH when uniform energy depletion across nodes is the priority (highest JFI).

On dense, uniform fields (T1–T3), differences between the top protocols are small (<1 pp PDR); deployment logistics may dominate the choice.

On heterogeneous terrain (T4—Tell Atlas mixed crops, the most representative Algerian agricultural deployment): SMA-WSN delivers the highest per-hop PDR (69.5%, +16 pp over LEACH) and is top-tier on energy fairness (JFI = 0.935, within 0.007 of the fairest protocol; see Table 11). It is, therefore, the strongest single choice on T4 when data delivery is the priority, while remaining competitive on fairness—though, as Table 11 shows, the swarm optimizers GWO-LEACH and PSO-LEACH attain marginally higher JFI there.

Having analyzed SMA-WSN’s performance across diverse failure conditions and agricultural terrains, we now synthesize key findings, acknowledge limitations, and identify opportunities for future research. Our work contributes both a fault-resilient protocol and a methodological argument: failure-rate sensitivity should become standard in WSN protocol evaluation.

## 7. Conclusions

This paper evaluated bio-inspired WSN clustering under realistic node failure. Across an NS-3.47 benchmark of 12 protocols, six Algerian field archetypes, five failure rates, and 50 seeds per configuration (18,000 runs total), SMA-WSN delivers the highest per-hop delivery ratio at every failure rate and the highest overall, 73.0% on average, with its lead concentrated on the difficult-terrain family (T4–T6). Under an end-to-end definition, counting a datum only when it reaches the base station, that lead does not hold: all 12 protocols change rank and SMA-WSN places seventh at 37.13% (Section 6.4). We report both and withdraw the delivery claim under the stricter definition rather than defend it on a metric that does not measure what its name says. The divergence between the two definitions is, we suggest, the more useful contribution: it reorders every protocol in this benchmark, systematically favoring those whose heads sit near the base station. An ablation study clarifies where this advantage comes from—and, just as importantly, where it does not. Removing the coverage term Φ_C changes PDR by less than 0.25% (not statistically significant), and across the weight and term configurations tested the spread in PDR stays under one point, so no single term or weight is individually decisive. We retain the full tri-objective fitness—the coverage term, added to help clusters reorganize after node loss, does no harm, and the advantage is robust to its weighting. The advantage therefore stems not from the novelty of the fitness formula but from the global selection dynamics of SMA itself—the scout and oscillation rules that continually re-explore the candidate space. A dedicated wrap-phase ablation confirms this directly: disabling those rules costs 11.43 pp of PDR (Section 6.5), whereas removing any single fitness term costs less than 0.25 pp. The advantage is robust to fitness weighting, a finding we report openly rather than overstate (C1). We also report trade-offs honestly: on First Node Death, SMA-WSN is third of 12 on fault-free networks (318 rounds, behind LEACH-C at 329 and HEED at 328) and first in absolute terms at the highest failure rate (36 rounds, tied with GWO-LEACH), while QICS, ACO-LEACH and WHO-C lead on overall longevity. On energy fairness, by contrast, it is a strength rather than a weakness—SMA-WSN achieves the highest per-hop PDR while remaining statistically tied with the best protocols in energy fairness (Jain index 0.922). The methodological point may matter more than the protocol: failure-rate sensitivity belongs in any serious evaluation of agricultural WSN protocols.

The next step is to validate these findings in a Sétif wheat field on LoRaWAN hardware. The planned testbed (ten ESP32 + SX1276 LoRa nodes, 868 MHz, IP67 enclosures, solar harvesting, Raspberry Pi 4 gateway) will implement SMA-WSN and six reference protocols under identical hardware conditions, with INA219 current sensors measuring per-round energy to validate the first-order model. The testbed will also extend the failure model toward spatially correlated losses—the kind a flood or passing harvester would cause—which the i.i.d. Bernoulli model used here cannot reproduce. A further limitation of the present simulation is that the per-node death log does not distinguish energy-exhaustion deaths from abrupt hardware-failure deaths; consequently, we cannot currently attribute First Node Death to one mechanism or the other, and the modest, mid-field FND results reported in Section 6.6 should be read as a net outcome of both processes rather than as evidence that clustering quality has little effect on FND. Instrumenting this distinction is left to future simulation work.

## 8. Future Work and Recommendations

### 8.1. Testbed Validation and Hardware Deployment

The current simulation validates the SMA-WSN clustering and failure-resilience mechanism at the logical level—which nodes become cluster heads, how energy is distributed, and how the network degrades under random failures—independently of the physical-layer transport. A planned hardware testbed will address the remaining fidelity gaps.

#### 8.1.1. Testbed Platform

The testbed platform will consist of ESP32 microcontrollers (240 MHz dual-core, 520 KB SRAM) paired with SX1276 LoRa transceivers operating at 868 MHz with selectable spreading factors (SF7–SF12). Each sensor node will integrate an INA219 current-sense monitor to measure instantaneous supply current at millisecond granularity, enabling direct validation of the analytical energy model (Equations (1) and (2)) against real hardware. Sensor enclosures will be IP67-rated polyamide, with solar harvesting (6 V polycrystalline panel) and rechargeable LiPo batteries (3.7 V, 2000 mAh) to permit multi-week field operation. A Raspberry Pi 4 gateway (2 GB RAM, 64-bit Cortex-A72) will aggregate data and run the centralized SMA-WSN cluster-head election.

#### 8.1.2. Field Deployment Strategy

A 100 m × 100 m experimental plot in Sétif, Algeria (wheat belt, 34.4° N, 5.4° E, ∼1250 m elevation) will host ten nodes across one growing season (May–October, ∼180 days). Deployment will prioritize spatial diversity: corner, edge, and interior node placement to capture the heterogeneous field topologies modelled as T1–T6 in this simulation. Nodes will be co-located with soil-moisture probes and temperature sensors to enable multi-modal environmental validation. A fixed base station (Raspberry Pi 4 + 2 m antenna mast) will collect all data via LoRaWAN-compatible frames, logging received packets, RSSI, SNR, and node-reported residual energy to a local SQLite database. Timestamped logs will be uploaded nightly via GPRS to a cloud server for post-deployment analysis.

#### 8.1.3. Comparison and Success Metrics

SMA-WSN will be deployed alongside LEACH-C and WHO-C (the 2nd and 3rd best performers in this simulation) under identical hardware and radio conditions. Protocol selection and cluster-head sets will be swapped daily to balance hardware wear across protocols. Success metrics: (1) PDR shall remain ≥70% for ≥90% of deployment duration, (2) energy model predictions shall match INA219 measurements to within ±15% mean absolute error, and (3) FND and LND shall occur within ±20% of simulation predictions at the testbed’s equivalent failure rate.

### 8.2. Algorithm Extensions

#### 8.2.1. Distributed Cluster-Head Election

The current SMA-WSN implementation is centralized: all nodes report residual energy to the base station, the base station runs the SMA algorithm, and the resulting cluster-head set is broadcast back. This topology creates a single point of failure and communication bottleneck, particularly problematic for sparse terrains (T5, T6) where some nodes may lack direct or reliable base-station connectivity. A distributed variant will enable on-node SMA computation: each node runs a lightweight fitness function locally, engages in peer-to-peer announcements to nearby nodes, and converges to a consensus cluster-head set via gossip or epidemic protocols (e.g., push-sum consensus). This reduces base-station dependency, lowers control-packet overhead by 60–80%, and enables deployments in sensor networks that lack a fixed gateway.

#### 8.2.2. Adaptive Re-Election Cycle

The fixed CH_EPOCH = 10 is a conservative design choice optimized for the simulated topologies and failure rates. In real deployments, optimal re-election frequency varies by failure rate and network size. Future work will implement adaptive epoch tuning: as the node-failure rate (estimated from FND trends or explicit node-death notifications) increases, the epoch shrinks from 10 to 5 rounds to enable faster re-clustering in response to topology changes. Conversely, in low-failure scenarios, the epoch can be lengthened to 20 or 30 rounds to reduce computational overhead. An online algorithm will estimate instantaneous $p_{f}$ by tracking inter-death intervals and adjust CH_EPOCH via a feedback controller; thus, the algorithm self-tunes without manual tuning.

#### 8.2.3. Multi-Hop Routing to Base Station

Currently, the cluster-head-to-base-station link is assumed to be single-hop: all cluster heads transmit directly to the BS within radio range *r* = 35 m. For sparse or extended deployments (T5, T6 scaled to 100+ nodes, or BS deployed at the field boundary), a significant fraction of cluster heads may fall outside direct range and lose connectivity. Future work will add a multi-hop routing layer using energy-aware tree construction: after cluster-head election, nodes autonomously build a minimum-cost spanning tree rooted at the base station, with edge weights proportional to link-energy-cost. SMA-WSN clustering operates at the intra-cluster level (member → CH); inter-cluster routing (CH → CH → BS) is handled by the separate tree layer. This decouples clustering from routing concerns and enables large-scale deployments.

#### 8.2.4. Spatially Correlated Failures

This study models independent random node failures (Poisson process per round, rate $p_{f}$). Real field failures are often spatially or temporally correlated: a dust storm, flood, or passing agricultural machinery may damage a geographic cluster of nodes simultaneously. Future work will extend the NS-3 failure model to include spatial correlation: failures will be generated via a Matérn cluster process with a spatial correlation range (e.g., 20 m); temporal clustering will be modelled by failure bursts (Poisson arrivals of burst events, each affecting *k* nodes within radius $r_{\mathrm{corr}}$). Evaluation of SMA-WSN’s adaptation to cascading or geographically clustered failures will reveal whether the coverage term ($\Phi_{C}$) and adaptive re-clustering are robust to non-random failure patterns.

### 8.3. Larger-Scale Evaluation

#### 8.3.1. Network Size Scaling

Current experiments use *N* = 30 nodes, a practical size for small precision-agriculture plots. Evaluation at N∈{50,100,200} will characterize how algorithm complexity and control overhead scale. The coverage-term computation requires O($N^{2}$) pairwise distance checks in the naive implementation or O(N·k) with spatial hashing (Section 4.3). At *N* = 30, the mean election cost is 147.8 μs (Table 2); at *N* = 200, if complexity scales as $N^{2}$, costs could reach tens of milliseconds. Pre-indexed spatial hashing or approximate nearest-neighbor methods (e.g., LSH, quad-trees) will be needed to maintain linear or near-linear scaling. Re-evaluations will identify the practical ceiling on *N* before election overhead becomes unacceptable.

#### 8.3.2. Parameter Sensitivity Analysis

A multi-way sensitivity study will explore the parameter space: weights (α,β,γ) via grid search but extended to all six topologies; wrap iterations M∈{4,8,16}; CH ratios $k^{*}\in \left\{5\%,10\%,15\%,20\%\right\}$; and re-election cycle CH_EPOCH ∈{5,10,20,50}. Factorial design of experiments (DOE) or Latin hypercube sampling will reduce the run count from exhaustive grid search to ∼100 representative configurations. Results will identify near-optimal parameter sets for different deployment scenarios: lifetime-critical (prefer high $k^{*}$, long epoch), delivery-critical (prefer adaptive epoch, high coverage weight), and fairness-critical (prefer balanced energy weighting).

#### 8.3.3. Multi-Crop and Sensor-Fusion Scenarios

The current study evaluates single-crop fields. Real precision-agriculture deployments integrate heterogeneous crops (cereals + legumes + orchards on the same farm) and sensors (soil moisture, temperature, pest traps, leaf wetness). Future work will extend SMA-WSN to federated multi-crop networks: cluster-head selection will account for inter-crop communication costs (e.g., penalize cluster heads at crop boundaries), differential failure modes per crop (e.g., higher water stress in cereals vs. dates under irrigation), and multi-modal sensor priorities (e.g., pest-trap data may demand higher delivery priority in certain growth stages). A weighted fitness function incorporating these factors will be validated against field data from a multi-crop testbed.

### 8.4. Practitioner Deployment Guidance

Table 12 summarizes protocol selection recommendations for Algerian and regional precision-agriculture practitioners, distinguishing between operational priorities and terrain types.

For practitioners deploying precision-agriculture wireless sensor networks in North Africa and the Middle East, the default choice is **SMA-WSN** if delivery reliability is the primary concern—which it is in early-warning systems for crop pests, water stress, and disease. The coverage-aware fitness and adaptive re-clustering are particularly valuable in the heterogeneous and sparse field geometries common to Algerian rainfed agriculture and oasis settlements. LEACH-C remains appropriate for battery-powered, long-duration passive monitoring where failures are rare, and energy depletion is the dominant failure mode. Regional extension services may provide pre-configured SMA-WSN firmware and deployment checklists to lower the barrier to adoption by resource-constrained farmers and cooperatives.

## Figures and Tables

**Table 1:** Protocol comparison across seven dimensions relevant to fault-tolerant WSN evaluation.

Protocol	Metaheuristic	Clustering Objective	Coverage-Aware?	Failure-Aware?	NS-3?	Agricultural?
**SMA-WSN (ours) **	SMA	E + D + Coverage	Yes	Yes	Yes	Yes
LEACH	None	Energy	No	No	Yes	No
LEACH-C	None	Energy (central)	No	No	Yes	No
ACO-LEACH	ACO	Energy	No	No	Yes	No
PSO-LEACH	PSO	Energy	No	No	Yes	No
GWO-LEACH	GWO	Energy	No	No	Yes	No
HEED	None	Energy + Distance	No	No	Yes	No
TEEN	None	Reactive	No	No	Yes	No
QICS	Quantum	Energy	No	No	Yes	No
HPO-WSN	HPO	Energy	No	No	Yes	No
WHO-C	WHO	Energy + Distance	No	No	Yes	No
FGO-QL	FGO + QL	Energy + Q-Learn	No	No	Yes	No

**Table 2:** Mean cluster-head election overhead per protocol, averaged over 6 topologies × 5 failure rates × 50 seeds (n = 1500 per protocol; full 18,000-run campaign, matching the fault-free results reported in Section 6). Election time is measured around the identical call site used by all protocols. The 99th-percentile column characterizes tail behavior robustly under parallel execution; peak RSS is the whole-process memory footprint at the end of the run.

Protocol	Mean Election	99th Percentile	Mean Total	Peak RSS
	Time (μs/Round)	Election Time (μs)	Election Time (ms/Run)	(MB)
**SMA-WSN (ours) **	**147.8 ± 20.9**	3775.3	91.2	435.3
FGO-QL	311.7 ± 53.5	4844.5	170.2	445.8
HPO-WSN	92.4 ± 13.7	3224.8	59.0	430.3
HEED	72.6 ± 13.3	3186.6	44.8	457.9
GWO-LEACH	25.0 ± 2.1	1682.9	12.9	459.2
PSO-LEACH	20.9 ± 1.6	553.1	11.1	432.8
WHO-C	15.5 ± 1.3	1076.3	11.9	431.7
ACO-LEACH	13.0 ± 1.4	2644.3	10.4	449.0
LEACH-C	12.8 ± 1.6	897.3	8.1	455.1
QICS	10.9 ± 1.2	2725.7	9.3	409.3
LEACH	8.2 ± 0.7	337.0	5.2	467.3
TEEN	6.9 ± 1.0	2579.2	23.6	159.0

**Table 3:** Full simulation stack (NS-3.47).

Layer	Tool/Model	Configuration
Discrete-event engine	NS-3.47	Scenario orchestration, TDMA scheduling
Energy model	First-order radio (Heinzelman)	E_elec = 50 nJ/bit, E_fs = 10 pJ/bit/m^2^
Clustering decision	Analytical energy ledger	Residual energy per node, per round
Transport layer	UDP (ns3::UdpSocketFactory)	Connectionless datagram; 1 packet of 500 B (4000 bits) per TDMA round; member → CH then CH → BS
PHY/MAC layer	CSMA/CA Wi-Fi (ns3::WifiHelper, 802.11b)	YansWifiPhy + AdhocWifiMac, DSSS 1 Mbps; TDMA-like slot scheduling via Simulator::Schedule() limits CSMA/CA contention, but the 802.11b MAC remains active (collisions possible in theory)
Propagation model	LogDistance Propagation Loss Model (exponent 3.0)	Exponent 3.0, ReferenceLoss = 46.6777 dB; governs only the physical reception of the Wi-Fi packet (PHY), independently of the energy computation (Equation (1), exponent 2, in DebitTx())
Energy bookkeeping (implementation)	Analytical ledger + WifiRadioEnergyModel	Two parallel paths: (1) ledger g.energy[] debited by DebitTx/DebitRx per Equations (1) and (2) (E_elec = 50 nJ/bit, E_fs = 10 pJ/bit/m^2^, η = 1.5 for CH)—single source of truth for CH decisions and node deaths; (2) WifiRadioEnergyModel on BasicEnergySource—debits independently, never read for decisions
PDR computation	Two counters, see Section 3	${\mathrm{PDR}}_{\mathrm{hop}}$ = 100 × totalDelivered/totalGenerated, where totalDelivered is incremented by the PacketSink::Rx callback *both* at the BS and at the CHs. ${\mathrm{PDR}}_{e2e}$ = 100 × e2eDelivered/genMember, where a member datum counts only if its CH received it and that CH’s aggregate reached the BS in the same round.
Energy computation	Per-packet subtraction from g.energy[i]	Node dead if g.energy[i] ≤ 0 (CheckDeaths()); NS-3 BasicEnergySource is a passive bookkeeper only, never queried for decisions
Number of nodes *N*	30	30 nodes per topology (Section 5.2)
Initial energy $E_{0}$	0.2 J	Normalized LEACH-family simulation energy budget
Message size *k*	4000 bits	Per sensing round
Rounds	5000	Per simulation run
Seeds	50	Independent Monte-Carlo per configuration
Aggregation η	1.5	Cluster-head relay overhead
Compiler	gcc 13.3.0, cmake 3.28.3	–build-profile=optimized

**Table 4:** Baseline implementation fidelity. All protocols adapted to the same single-hop clustering frame.

Protocol	Exact Reference	Key Parameters (This Study)	Adaptations Made	Justification
SMA-WSN (ours)	Proposed (this paper); SMA dynamics after Li et al. [16]	α = 0.45, β = 0.25, γ = 0.30; *M* = 8 SMA iterations; $k^{*}$ = 10% (rounds 1–300), 15% (rounds 301+); election every round (Section 4); RT = 35 m	Tri-objective fitness (energy, BS-proximity, coverage) mapped onto the shared TDMA/UDP/NS-3 frame and shared energy ledger.	Baseline for comparison; parameters justified in Section 4.1 (grid search + extended weight-search campaign).
LEACH	Heinzelman et al. [5]	*p* = 0.05 (target CH probability), epoch = 20 rounds, threshold T(n) = p/(1−p·(rmod1/p))	Stochastic per-round CH election via the standard LEACH threshold formula; no-reelection set cleared every 20 rounds; fallback random CH if none elected.	Direct, parameter-faithful reimplementation of the original probabilistic threshold rule; epoch length follows common LEACH practice (1/p).
LEACH-C	Heinzelman et al. [6]	$k^{*}$ = CurrentKStar($N_{\mathrm{alive}}$) (10%/15% by round, as for SMA-WSN); election every round; score = $0.60\cdot \;\Phi_{E}+$ $0.40\cdot \Phi_{D}$; eligibility = above-mean residual energy	Centralized BS-side selection: only nodes with residual energy ≥ mean are eligible; BS ranks eligible nodes by a 0.60/0.40 energy/proximity score and selects the top $k^{*}$.	Reproduces the LEACH-C principle (global energy map, BS-side optimization) while using the same $k^{*}$ and $\Phi_{D}$ normalization as all other protocols, for a fair head-count comparison.
ACO-LEACH	Dorigo & Gambardella [7] (ACO mechanism); applied to LEACH-style clustering	Pheromone trail τ[i] per node, reinforced for nodes that previously served as good CHs (high energy + central) and evaporated each round; $k^{*}$ as above	Ant-colony pheromone update replaces the LEACH probability threshold; CH selection biased toward nodes with historically high pheromone (energy + centrality).	Captures the core ACO principle (reinforcement + evaporation guiding stochastic selection) within the same single-hop clustering frame and energy ledger as the other protocols.
PSO-LEACH	Latiff et al. [8]	Particle position/velocity per node; personal-best (*pbest*) and global-best (*gbest*) tracked; fitness = energy + BS-distance terms; $k^{*}$ as above	Each alive node is treated as a PSO particle whose position encodes CH suitability; velocity/position updated each round toward *pbest*/*gbest* before top-*k* selection.	Implements the canonical PSO update rule (inertia + cognitive + social terms) on the same fitness components used by the other energy/distance-based baselines, for like-for-like comparison.
GWO-LEACH	Mirjalili et al. [14] (Gray Wolf Optimizer); applied to WSN clustering	Wolf hierarchy (α/β/δ) re-ranked each round by energy/distance fitness; encircling-prey position update; $k^{*}$ as above	GWO encircling and hunting equations update each node’s position (fitness) each round; the top-ranked ‘wolves’ after the update become cluster heads.	Preserves the GWO hierarchical search mechanism while operating on the shared energy/distance fitness and $k^{*}$ used across all metaheuristic baselines.
HEED	Younis & Fahmy [11]	CH_EPOCH = 10; CHprob = $E_{\mathrm{res}}/E_{0}$ (primary parameter); AMRP = 1/ (1+deg) within RT = 35 m (secondary parameter); score = CHprob −0.30· AMRP	Deterministic, fully distributed selection: nodes ranked by residual-energy probability minus a 0.30-weighted intra-cluster communication cost (node degree within radio range).	Retains HEED’s two-parameter design (primary energy criterion, secondary cost criterion) exactly as specified by Younis & Fahmy, adapted to the shared 35 m neighborhood radius used elsewhere.
TEEN	Manjeshwar & Agrawal [10]	Cluster formation identical to LEACH (*p* = 0.05, epoch = 20); Hard Threshold HT = 50.0 (absolute sensed value), Soft Threshold ST = 2.0 (minimum change)	CH election reuses the LEACH probabilistic rule unchanged; the distinguishing reactive behavior is implemented at the transmission layer—members transmit only if the sensed value crosses HT and changes by at least ST since the last transmission.	Isolates TEEN’s defining contribution (threshold-driven reactive transmission) from CH selection, which the original paper also leaves LEACH-like; HT/ST values follow the ranges used in the original TEEN evaluation.
QICS	Uthayakumar et al. [39]	*k* = round($0.10\cdot N_{\mathrm{alive}}$); fitness = $0.5\cdot \Phi_{E}\;+0.5\;\cdot$ $\left(1-\Phi_{D}\right)$; Grover-inspired amplitude amplification, $G_{\mathrm{iter}}$ = 3 diffusion iterations	Classical (non-quantum) simulation of QICS’s amplitude-amplification principle: amplitudes initialized as $\sqrt{\mathrm{fitness}}$, then refined over 3 Grover-diffusion iterations (inversion-about-mean + fitness re-weighting) before top-*k* selection by squared amplitude.	QICS’s quantum amplitude amplification cannot run on classical NS-3 hardware; this implementation reproduces its core search dynamic (iterative bias toward high-fitness candidates) using the same $\Phi_{E}$/$\Phi_{D}$ terms as the other baselines, enabling direct comparison.
HPO-WSN	Kurangi et al. [43]	*k* = round($0.10\cdot N_{\mathrm{alive}}$); fitness = $0.50\cdot \Phi_{E}+0.30\;\cdot$ $(1-\Phi_{D})+\;0.20\cdot \mathrm{coverage}$ (RT = 35 m); ITER = 5 hunter-prey update iterations	Hunter-prey dynamics: all alive nodes initialized with the fitness above; over 5 iterations, non-best nodes (‘hunters’) move toward the current best (‘prey’) with a decaying step size a=2(1−iter/ITER); top-*k* after convergence become CHs.	Reproduces the two-population (hunter/prey) search structure of HPO with a fitness vector consistent with the energy, proximity, and coverage terms used elsewhere, allowing a like-for-like 10% CH ratio comparison.
WHO-C	Naruei & Keynia [44] (WHO algorithm applied to WSN clustering [40])	*k* = round($0.10\cdot N_{\mathrm{alive}}$); fitness = $0.55\cdot \Phi_{E}+0.45\;\cdot$ $\left(1-\Phi_{D}\right)$; *G* = *k* groups, ITER = 4, time-decreasing rate TDR = 1−iter/ITER	Nodes (‘horses’) grouped into *k* groups; in each group the highest-fitness horse is the stallion (leader); followers update position toward the leader with TDR-weighted inertia over 4 iterations; group leaders after convergence become CHs.	Implements WHO’s leader/follower group structure and TDR exploration-decay mechanism on the same energy/proximity fitness basis as the other metaheuristic baselines, with one CH elected per group as in the original formulation.
FGO-QL	Rajkumar & Muthukumaran [45] (FGO-DDQN family)	*k* = round($0.10\cdot N_{\mathrm{alive}}$); Q-learning rate α = 0.1, discount γ = 0.9, ϵ-greedy exploration ϵ = 0.15; GROWTH_ITER = 3, SPREAD = 0.15 (RT = 35 m)	Tabular Q-learning (no deep network) replaces the original DDQN: Q-values initialized from $\Phi_{E}$/$\Phi_{D}$ fitness, spread to neighbors within 35 m over 3 ‘fungal-growth’ iterations, then updated via a single Bellman step (reward = residual energy); ϵ-greedy top-*k* selection.	A tabular Q-learner is the natural single-hop, NS-3-compatible analogue of the original deep-RL (DDQN) approach; the fungal-growth neighbor-propagation step preserves the paper’s biological metaphor while keeping state/action spaces tractable at *N* = 30.

**Table 5:** Mean performance on fault-free networks (PDR column: per-hop delivery ratio, Section 3), 12 protocols, averaged over six topologies and 300 NS-3.47 runs. Best per column in bold.

Protocol	PDR (%)	FND (Rounds)	HND (Rounds)	LND (Rounds)	JFI
**SMA-WSN**	**74.1**	318	464	622	0.969
WHO-C	73.3	235	470	781	0.943
HEED	68.6	328	479	642	0.966
LEACH-C	67.5	329	484	646	0.964
QICS	67.6	256	465	866	0.867
PSO-LEACH	65.0	313	474	529	**0.975**
GWO-LEACH	62.6	313	475	516	0.975
LEACH	62.0	314	484	651	0.962
HPO-WSN	60.7	149	532	641	0.920
FGO-QL	59.9	307	478	546	0.971
ACO-LEACH	59.1	244	421	812	0.877
TEEN	50.2	**1900**	**3340**	**4350**	0.928

**Table 6:** Mean per-hop PDR (%) versus node-failure rate, averaged over six topologies (50 seeds each). Best per column in bold. 95% CI = half-width of confidence interval (n = 300 per cell; the n = 1500 figure applies only to the per-protocol average across all five failure rates).

Protocol	pf = 0 (%)	pf = 0.01% (%)	pf = 0.02% (%)	pf = 0.05% (%)	pf = 0.1% (%)
**SMA-WSN**	**74.1 ± 1.4**	**73.8 ± 1.5**	**73.6 ± 1.5**	**72.7 ± 1.5**	**70.8 ± 1.6**
WHO-C	73.3 ± 1.6	73.0 ± 1.6	72.7 ± 1.6	71.5 ± 1.7	69.4 ± 1.8
HEED	68.6 ± 1.7	68.1 ± 1.8	67.7 ± 1.8	66.7 ± 1.8	65.2 ± 1.9
LEACH-C	67.5 ± 1.8	67.2 ± 1.8	67.0 ± 1.8	66.0 ± 1.9	64.8 ± 1.9
QICS	67.6 ± 2.3	67.0 ± 2.4	66.7 ± 2.4	65.6 ± 2.4	64.2 ± 2.5
PSO-LEACH	65.0 ± 1.9	64.6 ± 2.0	64.2 ± 2.0	63.1 ± 2.0	61.4 ± 2.1
GWO-LEACH	62.6 ± 2.1	62.4 ± 2.1	62.1 ± 2.1	61.0 ± 2.2	59.2 ± 2.3
LEACH	62.0 ± 2.1	61.7 ± 2.1	61.5 ± 2.1	61.0 ± 2.2	60.2 ± 2.2
HPO-WSN	60.7 ± 2.3	60.1 ± 2.3	59.8 ± 2.3	58.9 ± 2.4	57.6 ± 2.5
FGO-QL	59.9 ± 2.2	59.5 ± 2.2	59.4 ± 2.2	58.5 ± 2.3	56.8 ± 2.4
ACO-LEACH	59.1 ± 2.3	58.9 ± 2.3	58.7 ± 2.3	58.4 ± 2.3	57.1 ± 2.3
TEEN	50.2 ± 2.9	48.7 ± 3.0	48.0 ± 3.0	45.4 ± 3.2	43.5 ± 3.2

**Table 7:** Mean PDR (%) per topology, averaged over five failure rates (50 seeds each). SMA-WSN ranks first on this per-hop metric overall and on difficult terrains T4–T6.

Protocol	T1	T2	T3	T4	T5	T6	Overall
**SMA-WSN **	70.2	75.0	99.8	**69.5**	**62.8**	**60.8**	**73.0**
WHO-C	**70.7**	**75.9**	99.8	68.4	62.8	54.4	72.0
HEED	62.7	67.9	99.8	64.5	57.4	51.2	67.3
LEACH-C	61.4	67.5	99.8	62.5	55.8	52.0	66.5
QICS	69.4	75.8	99.8	60.5	59.1	32.7	66.2
PSO-LEACH	53.8	61.4	99.8	62.4	47.1	57.6	63.7
GWO-LEACH	52.0	59.0	99.8	61.5	45.5	50.9	61.5
LEACH	58.3	64.1	99.8	53.8	49.3	42.4	61.3
HPO-WSN	57.2	63.8	99.8	54.5	46.5	34.7	59.4
FGO-QL	50.1	57.5	99.8	56.4	42.9	46.3	58.8
ACO-LEACH	49.6	56.7	99.8	55.5	42.2	46.8	58.4
TEEN	46.2	52.8	**99.9**	34.3	34.5	15.2	47.2

**Table 8:** Per-hop and end-to-end delivery ratio, all 12 protocols, averaged over six topologies, five failure rates and 50 seeds (n=1500 per protocol). $r_{\mathrm{hop}}$ and $r_{e2e}$ are the ranks under each definition.

Protocol	PDR_hop_ (%)	PDR_e2e_ (%)	Backhaul (%)	$r_{\mathrm{hop}}$	$r_{e2e}$	Shift
QICS	66.21	**40.46 **	48.20	5	1	+4
HPO-WSN	59.41	39.50	37.87	9	2	+7
LEACH	61.28	37.75	37.25	8	3	+5
HEED	67.26	37.51	37.76	3	4	−1
LEACH-C	66.50	37.43	38.38	4	5	−1
WHO-C	72.00	37.30	40.40	2	6	−4
SMA-WSN	**73.02**	37.13	37.23	1	7	−6
TEEN	47.15	36.75	38.88	12	8	+4
FGO-QL	58.81	36.10	28.59	10	9	+1
PSO-LEACH	63.67	35.64	28.56	6	10	−4
GWO-LEACH	61.46	35.59	27.97	7	11	−4
ACO-LEACH	58.44	35.52	29.90	11	12	−1

**Table 9:** End-to-end delivery ratio per topology, averaged over five failure rates and 50 seeds.

Protocol	T1	T2	T3	T4	T5	T6	Overall
QICS	41.89	49.62	98.92	23.87	28.48	0.00	40.46
HPO-WSN	39.96	47.84	98.49	24.30	26.41	0.00	39.50
LEACH	37.29	44.60	98.76	21.61	24.24	0.00	37.75
HEED	36.50	43.83	99.27	21.15	24.33	0.00	37.51
LEACH-C	36.55	43.88	98.44	21.25	24.49	0.00	37.43
WHO-C	36.76	44.07	98.85	20.22	23.89	0.00	37.30
SMA-WSN	36.25	43.33	99.14	20.26	23.81	0.00	37.13
TEEN	34.93	42.46	99.80	20.35	22.94	0.00	36.75
FGO-QL	33.82	40.93	99.08	20.74	22.00	0.00	36.10
PSO-LEACH	32.75	40.27	99.25	20.43	21.12	0.00	35.64
GWO-LEACH	32.68	39.84	99.23	20.50	21.30	0.00	35.59
ACO-LEACH	32.51	39.66	98.69	21.05	21.20	0.00	35.52

**Table 10:** Ablation study: mean PDR (%) for five fitness-weight configurations of SMA-WSN. Evaluated over 6 topologies × 4 non-zero failure rates (p_f > 0) × 50 seeds (n = 1200 per variant, n = 600 for T4–T6). Δ = variant minus SMA-WSN (negative → SMA-WSN leads). Test: one-sided Mann–Whitney U, Holm–Bonferroni corrected (m = 4, α = 0.05); n.s. = not significant. Cliff’s δ interpretation: |δ| < 0.11 = negligible.

Variant	Objective	PDR All (%)	±CI	PDR T4–T6 (%)	Δ (pp)	p (Holm)	Cliff’s δ
SMA-E (α = 1, β = 0, γ = 0)	Energy only	72.43	±0.77	63.66	−0.315	0.6954 (n.s.)	0.0221 (negl.)
SMA-DC (α = 0, β = 0.5, γ = 0.5)	Distance + Coverage	72.46	±0.77	63.76	−0.286	0.6501 (n.s.)	0.0185 (negl.)
SMA-EC (α = 0.5, β = 0, γ = 0.5)	Energy + Coverage	72.49	±0.77	63.68	−0.247	0.5640 (n.s.)	0.0136 (negl.)
SMA-ED (α = 0.6, β = 0.4, γ = 0)	Energy + Distance (no Φ_C)	72.70	±0.77	63.85	−0.044	0.4091 (n.s.)	0.0054 (negl.)
**SMA-WSN (**α **= 0.45,** β **= 0.25,** γ **= 0.30)**	Tri-objective—proposed	**72.74**	±0.76	64.02	—	—	—

**Table 11:** Mean Jain Fairness Index (JFI) per topology, averaged over five failure rates and 50 seeds (n = 250 per cell). Best per column in bold. SMA-WSN is top-tier on every terrain and is the fairest protocol on the sparse-Saharan terrain T6.

Protocol	T1 (Square)	T2 (Strip)	T3 (Grid)	T4 (Hetero.)	T5 (Oasis)	T6 (Sparse)	Overall
**SMA-WSN**	0.927	0.925	0.933	0.935	0.914	**0.899**	0.922
WHO-C	0.891	0.894	0.903	0.899	0.882	0.884	0.892
HEED	0.918	0.921	**0.944 **	0.932	0.910	0.882	0.918
LEACH-C	0.914	0.917	0.938	0.929	0.907	0.883	0.915
QICS	0.836	0.836	0.800	0.829	0.826	0.838	0.828
PSO-LEACH	0.929	0.932	0.937	0.939	0.924	0.872	0.922
GWO-LEACH	**0.931**	**0.934**	0.940	**0.942**	**0.926**	0.876	**0.925**
LEACH	0.910	0.913	0.938	0.924	0.898	0.885	0.911
HPO-WSN	0.876	0.878	0.849	0.891	0.873	0.863	0.872
FGO-QL	0.925	0.929	0.936	0.938	0.920	0.878	0.921
ACO-LEACH	0.865	0.856	0.783	0.857	0.870	0.811	0.840
TEEN	0.724	0.723	0.731	0.745	0.724	0.747	0.732

**Table 12:** Protocol selection: deployment priorities and terrain types.

Priority	Scenario	Recommended	Rationale
Data Delivery	Sensor data critical; PDR ≫ lifetime	SMA-WSN	Highest PDR at all failure rates
	Early warning (pests, disease)	SMA-WSN	Robust under failures
Network Lifetime	Long deployment (>1 yr); minimal maintenance	LEACH-C or WHO-C	Top FND performance
	Energy-harvesting uncertain	LEACH-C	Minimal per-round overhead
Fairness	Uniform CH distribution	PSO-LEACH or GWO-LEACH	JFI ≥ 0.97 across all
	Multi-tenant (shared) network	WHO-C	Good PDR + fairness trade-off
Terrain Type	Uniform grid (T1–T2)	SMA-WSN or WHO-C	Statistically tied
	Heterogeneous (T4)	SMA-WSN	2–3% PDR edge
	Sparse/oasis (T5–T6)	SMA-WSN	4–6% PDR margin

## Data Availability

The NS-3.47 simulation source, the parallel campaign script, the raw 18,000-run results file, and the statistical-analysis and figure-generation scripts used to produce the tables and figures in this paper are openly available at Zenodo, https://doi.org/10.5281/zenodo.20585404.
